# Novel Anthranilic Acid Hybrids—An Alternative Weapon against Inflammatory Diseases

**DOI:** 10.3390/ph16121660

**Published:** 2023-11-29

**Authors:** Miglena Milusheva, Mina Todorova, Vera Gledacheva, Iliyana Stefanova, Mehran Feizi-Dehnayebi, Mina Pencheva, Paraskev Nedialkov, Yulian Tumbarski, Velichka Yanakieva, Slava Tsoneva, Stoyanka Nikolova

**Affiliations:** 1 Department of Organic Chemistry, Faculty of Chemistry, University of Plovdiv, 4000 Plovdiv, Bulgaria or miglena.milusheva@mu-plovdiv.bg (M.M.); minatodorova@uni-plovdiv.bg (M.T.); 2 Department of Bioorganic Chemistry, Faculty of Pharmacy, Medical University of Plovdiv, 4002 Plovdiv, Bulgaria; 3 Department of Medical Physics and Biophysics, Faculty of Pharmacy, Medical University of Plovdiv, 4002 Plovdiv, Bulgaria; vera.gledacheva@mu-plovdiv.bg (V.G.); iliyana.stefanova@mu-plovdiv.bg (I.S.); mina.pencheva@mu-plovdiv.bg (M.P.); 4 Department of Chemistry, Faculty of Science, University of Sistan and Baluchestan, Zahedan P.O. Box 98135-674, Iran; m.feizi@sutech.ac.ir; 5 Department of Pharmacognosy, Faculty of Pharmacy, Medical University of Sofia, 1000 Sofia, Bulgaria; pnedialkov@pharmfac.mu-sofia.bg; 6 Department of Microbiology, Technological Faculty, University of Food Technologies, 4002 Plovdiv, Bulgaria; tumbarski@abv.bg (Y.T.); yanakieva_vili@abv.bg (V.Y.); 7 Department of Analytical Chemistry and Computer Chemistry, University of Plovdiv, 4000 Plovdiv, Bulgaria; slava.tsoneva@uni-plovdiv.bg

**Keywords:** synthesis, hybrid molecules, anthranilic acid, in silico, DFT calculations, docking, anti-inflammatory, spasmolytic activity, antimicrobial

## Abstract

Anti-inflammatory drugs are used to relieve pain, fever, and inflammation while protecting the cardiovascular system. However, the side effects of currently available medications have limited their usage. Due to these adverse effects, there is a significant need for new drugs. The current trend of research has shifted towards the synthesis of novel anthranilic acid hybrids as anti-inflammatory agents. Phenyl- or benzyl-substituted hybrids exerted very good anti-inflammatory effects in preventing albumin denaturation. To confirm their anti-inflammatory effects, additional ex vivo tests were conducted. These immunohistochemical studies explicated the same compounds with better anti-inflammatory potential. To determine the binding affinity and interaction mode, as well as to explain the anti-inflammatory activities, the molecular docking simulation of the compounds was investigated against human serum albumin. The biological evaluation of the compounds was completed, assessing their antimicrobial activity and spasmolytic effect. Based on the experimental data, we can conclude that a collection of novel hybrids was successfully synthesized, and they can be considered anti-inflammatory drug candidates—alternatives to current therapeutics.

## 1. Introduction

Inflammation and pain have been the primary focus of international scientific research since they are implicated in almost all human and animal diseases [1]. Inflammatory diseases occur from the body’s response to tissue damage. Acute inflammation that fails to be regulated leads to chronic inflammation, predisposing the body to the deployment of cancer, neurodegenerative diseases, or autoimmune diseases [2,3]. Leucocytes, macrophages, and mast cells undergo various biological responses, releasing signaling chemicals that are involved in the complex pathophysiological process of inflammation. Inflammatory mediators generated include nitric oxide (NO), prostaglandin (PGE2), and tumor necrosis factor (TNF-α), as well as phagocytic uptake [4,5]. These factors cause the extravasation of fluids, proteins, and leucocyte buildup at the site of inflammation, which results in the formation of edema [6]. Furthermore, it is broadly accepted that cytokines, which are produced by immunological or central nervous system cells, may directly sensitize the peripheral nociceptors [7]. By influencing the transducing ability of free nerve terminals, bradykinins, TNF, interleukins (ILs), and prostaglandins (PGs) cause hyperalgesia and pain. Fever is caused by an infection that produces pyrogens like ILs, TNF-, and interferon, which cause the hypothalamus to produce PGE2 and raise its internal temperature. Fever or pyrexia is a subsequent result of inflammation [8]. Fever, discomfort, and inflammation are all linked to increased prostaglandin production [9]. As a result, most anti-inflammatory drugs are anticipated to have analgesic and antipyretic properties [10,11]. Inflammation is related to cancer as well. Inflammation has a significant impact on tumor development and proliferation [12].

The gut microbiota plays a key role in the development of gastrointestinal diseases, and recently the relationship between the gut microbiota and health has become increasingly obvious [13,14]. The microbiota diversity and abundance maintain an equilibrium known as eubiosis, which is important and necessary for human well-being. Age, stress, antibiotics, poor nutrition, and other factors can all contribute to dysbiosis, which can cause inflammation and advance chronic diseases. Gut dysbiosis is followed by chronic inflammatory bowel disease, such as Crohn’s disease and ulcerative colitis. Low bacterial diversity and the altered synthesis of short-chain fatty acids are two changes in the microbiota profile that have been noticed [14,15].

Non-steroidal anti-inflammatory drugs are used to relieve pain, fever, and inflammation while also protecting the cardiovascular system. However, the side effects of currently available anti-inflammatory medications, which include gastric ulcer, renal damage, bronchospasm, and cardiac problems, have limited their usage [16,17,18]. Due to the adverse effects of non-steroidal anti-inflammatory drugs and opioids, there is a high demand for the search of new drugs with fewer or no side effects.

**Figure 1:**
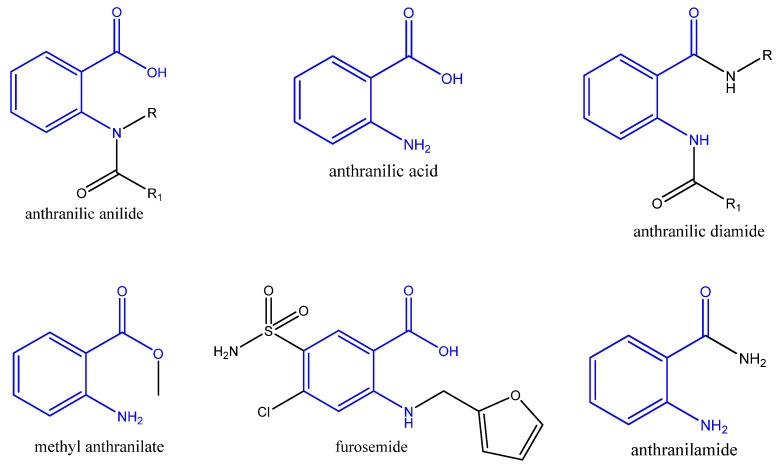
Structure of anthranilic acid, its derivatives and a diuretic furosemide. (The anthranilic acid residue is in blue).

Anthranilic acid and its analogs, on the other hand, are important components in several bioactive compounds and drugs, with a variety of biological activities in the treatment of various diseases (Figure 1). The anthranilic acid derivatives have antibacterial, antiviral, and insecticidal capabilities, as well as having potential uses as P-glycoprotein inhibitors for controlling drug resistance in cancer cells. Anthranilic acid derivatives have shown very good anti-inflammatory, analgesic, and antipyretic activity and no toxicity [19]. The amides of anthranilic acid are used in the treatment of numerous metabolic issues, as well as anti-inflammatory drugs. Anthranilic acid itself has a remarkable biological profile. Its structural nucleus has undergone extensive research for the design of medications intended to modulate the biochemical and metabolic pathways involved in the pathogenesis of numerous disorders [20,21,22,23]. Anthranilic acid diamides have uses as P-glycoprotein inhibitors to control drug resistance in cancer cells; as well as this they can serve as cholecystokinin receptor antagonists [24]. One well-known diuretic furosemide belongs to the anthranilic acid derivatives (Figure 1).

**Figure 2:**
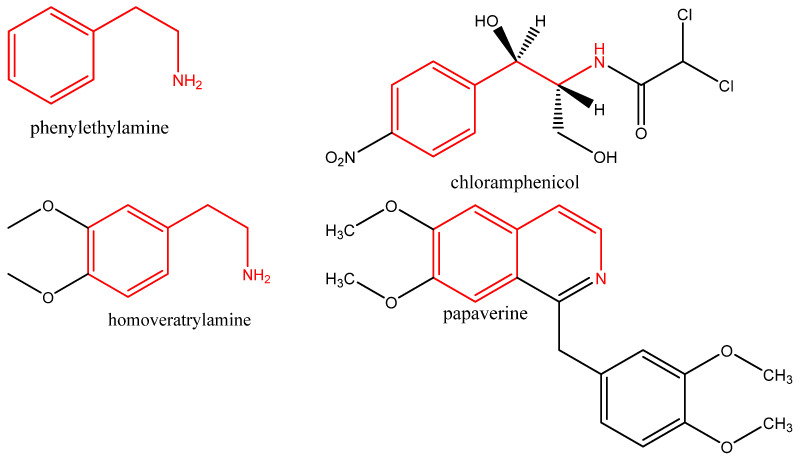
Chemical structures of phenylethylamine, homoveratrylamine, chloramphenicol, and papaverine. (2-phenylethylamine residue is in red).

Homoveratrylamine, on the other hand, belongs to the 2-phenylethylamine class. It is a dopamine-based analog of mivacurium chloride that is used as a pharmacological agent. Its derivatives can also be used as antiarrhythmic agents [25]. One isoquinoline with homoveratrylamine residue is papaverine. It has been used for decades as a muscle relaxant, brain and coronary vasodilator, and non-specific spasmolytic agent [26] (Figure 2). A phenylethylamine derivative, chloramphenicol, is used to treat and manage superficial eye infections such as bacterial conjunctivitis and otitis externa (Figure 2) [27,28].

Finding new therapeutic strategies, manipulating established pharmacological targets, or validating novel agents with a different mechanism of action remain the top priority. Based on the pharmacological activities of anthranilic acid derivatives and homoveratrylamine and in continuation of previous work [20], the current trend of research has shifted towards the synthesis of novel anthranilic acid hybrids as novel anti-inflammatory drug candidates.

## 2. Results and Discussion

### 2.1. Synthesis of Hybrid Molecules

**Scheme 1:**
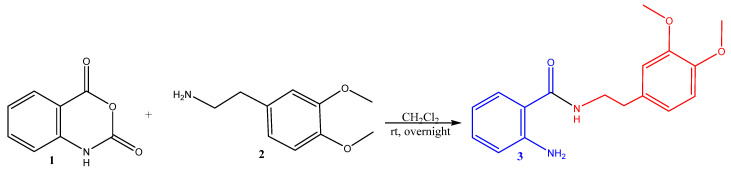
Synthesis of the starting hybrid 2-amino-N-(3,4-dimethoxyphenethyl)benzamide **3**. (Anthranilic acid residue is in blue; 2-phenylethyl moiety is in red).

Our synthetic strategy was based on the ring opening of isatoic anhydride **1** with homoveratrylamine **2** to form the hybrid molecule **3** with a high purity and yield (99%), according to the following (Scheme 1).

The structure of the resulting compound was verified using spectral data. FT-IR, ^1^H-NMR, ^13^C-NMR, and MS-spectra (Supplementary Materials Figures S1–S4) were completely consistent with the corresponding molecule.

**Scheme 2:**
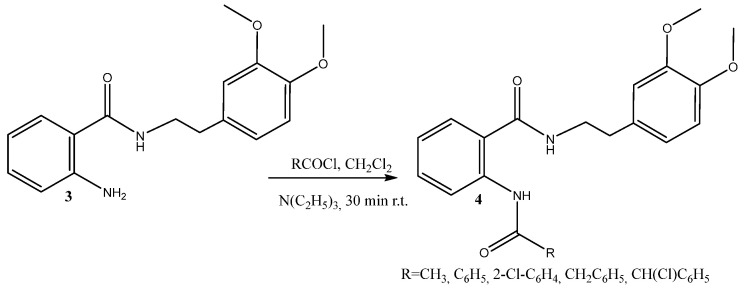
Synthesis of the diamides **4a**–**e**.

Amide synthesis is extremely important because of the presence of the amide group in biological systems and its crucial significance in medicinal chemistry. Amide groups are prominent in biological systems and play an important role in biological systems. The most often employed amide-forming techniques in the pharmaceutical sector [29] are essential for producing the structural core of peptides, proteins, and other macromolecules [30]. Amide interactions with biological targets are a crucial consideration in the synthesis of novel medicines [31]. A quarter of all commercially available drugs and many therapeutic prospects have an amide bond [32]. To achieve novel diamides, we applied the newly synthesized hybrid **3** in an acylation reaction (Scheme 2).

Reaction with acyl chlorides worked well and efficiently furnished the desired diamides **4a**–**e** in 78–82% yield (Table 1). Functional groups, including phenyl and benzyl substituents, were well tolerated because of the previously found activity [33].

The resultant compounds were characterized by their melting point, IR, ^1^H, ^13^C-NMR, and HRESIMS spectra. Spectral data confirmed the structure of all the obtained compounds (Supplementary Materials Figures S5–S24). In ^1^H-NMR, the broad singlet of the amine group at 5.45 ppm is shifted to the region 11.05–12.13 ppm due to the formation of the new amide group in **4a**–**e**, as well as a signal for the new carbonyl group of the second C=O in ^13^C-NMR.

### 2.2. In Silico Predictions

The systematic use of experiments, a necessary step for the development of novel drugs, is constrained by a variety of reasons. The prevalence of newly synthesized compounds, the quantitative limitations of tissue samples, and the need to limit animal testing are just a few reasons to mention. In this context, it is plausible to assume that biological investigations might be augmented and even entirely replaced by in silico computer models, which are both a good supplement and a viable substitute for biological investigations [34]. For this reason, we made the decision to incorporate several software products into our ongoing investigation into novel hybrid compounds as prospective drug candidates. A drug candidate needs to reach its pharmacological target within the body, get to the right concentration at the site of action, and stay there long enough to be utilized as a medicine. Due to their poor pharmacokinetics and bioavailability, many promising biologically active compounds that are intended for use as medications fail. In our calculations, the computer program SwissADME was used to identify and discard any compounds violating the rules to qualify as a drug; the Prediction of Activity Spectra for Substances (PASS Online Program) was employed in our estimations of the anticipated biological effects [35,36,37]. The PASS predicted the potential anti-inflammatory activity, as well as spasmolytic activity, for the target compounds, which are a permanent interest for our studies. The predicted LD_50_ (ProTox-II) for all the compounds is between 1000 mg/kg and 2000 mg/kg and none of the predicted compounds showed organ toxicity, cytotoxicity, or hepatotoxicity.

In silico ADME studies, also known as computer-based studies, play a crucial role in the drug discovery and development process. ADME stands for Absorption, Distribution, Metabolism, and Excretion, which are key factors that determine the pharmacokinetics and efficacy of a drug. In silico ADME studies involve the use of computational models and algorithms to predict the ADME properties of a drug, which not only save time and resources but also help in the identification of potential safety concerns. In the early phase of drug development, ADMET analysis is very helpful in facilitating a considerable decrease in unsuccessful clinical trials [38]. Molecular properties such as the partition coefficient (Log P o/w), molecular weight, hydrogen bond donors and acceptors, topological polar surface area (TPSA), and violation of the Lipinski rule of five were assessed (Table 2).

When creating new drugs, the drug likeness features outlined in Lipinski’s Rule of Five are necessary [39]. According to the rule, at least two parameters from four basic pharmacokinetic properties (MW ≤ 500, XLOGP3 ≤ 5, the number of hydrogen bond donors ≤ 5, and hydrogen bond acceptors ≤ 10.6) should be fulfilled for drug candidates. In terms of bioavailability (BA) [40], an optimal range of distinct properties have been reported involving lipophilicity (XLOGP3: −0.7 to +5.0), size (MW: 150 to 500 g/mole), polarity (TPSA: 20 to 130 Å^2^), ESOL or estimated solubility (log S: not more than 6), saturation (Fraction Csp^3^ or fraction of carbons in the sp^3^ hybridization: not less than 0.25), and flexibility (RB: no more than 9).

Finally, Lipinski’s Rule of Five, a widely used rule in drug discovery to assess the drug-like properties of a compound, was performed to study our compounds. It is recommended that an orally active drug candidate should not have more than one violation of Lipinski’s rule. As shown in Table 2, all compounds tested met the established criteria.

The absorption of a drug is a critical factor in determining its efficacy and safety. It refers to the process by which a drug enters the bloodstream and reaches its target site of action. Understanding the factors that influence drug absorption is essential for optimizing drug therapy and minimizing adverse effects. Because of this, the absorption of the drug was evaluated based on aqueous solubility, intestinal absorption and permeability. The ADME investigation showed that all the compounds cross through the BBB and present good gastrointestinal absorption, and could be considered as a drug candidate according to Lipinski’s rule of five [39], an important step in a drug discovery process. The calculated TPSA values of the compounds were in the range of 73.58–76.66 Å^2^, indicating high gastrointestinal absorption and BBB penetration. The low number of rotatable bonds for the **3** and **4a** compounds corresponds with sufficient oral bioavailability. The solubility parameter is very important for the pharmaceutical applications of the drug. All drugs were predicted by SwissADME to be water-soluble or moderately soluble in water. The values of the drug likeness parameters of all compounds were found to remain within the BA, which confirmed that all the synthesized compounds behave as “drug-like” substances. All of the compounds were calculated to have a BA score of 0.55, which demonstrates good oral bioavailability, according to Lipinski’s rule [39]. Moreover, they had good synthetic accessibility (SA) scores, which are considerable parameters during the drug discovery processes. All the compounds were predicted to have good skin permeability. Compounds **3** and **4a** had the lowest skin permeability with log Kp −6.02 and −6.38. The log Kp coefficients of the other compounds were between −5.35 and −5.71.

Additional factors to consider in drug development include metabolite formation mediated by cytochrome P450 (CYP) enzyme activities and drug clearance. CYP450 enzymes are a family of heme-containing enzymes that are involved in the metabolism of a wide range of endogenous compounds and xenobiotics. These enzymes play a crucial role in drug metabolism, and their activity can influence the efficacy and safety of many therapeutic agents. CYP2C9, for example, is primarily expressed in the liver and plays a critical role in the metabolism of numerous drugs. The novel hybrids were studied as possible CYP2D6, CYP3A4, CYP1A2, CYP2C19, and CYP2C9 enzyme inhibitors. It is noteworthy that all compounds showed the ability to inhibit CYP2D6, CYP3A4, CYP2C19, and CYP2C9 enzymes. These results clearly indicate that all these compounds show appropriate pharmacokinetic properties and after additional tests can serve as new therapeutic agents.

### 2.3. Biological Evaluation of the Hybrid Molecules

#### 2.3.1. Anti-Inflammatory Activity

##### In Vitro Inhibition of Albumin Denaturation

Modern theories suggest that inflammation is a process that occurs in response to an injury or illness. An anti-inflammatory quality of a drug or treatment is the capacity to reduce swelling or inflammation. Anti-inflammatory drugs reduce pain by lowering inflammation. Steroidal anti-inflammatory drugs and non-steroidal anti-inflammatory drugs are two kinds of medications that are frequently used to treat inflammation. Non-steroidal ones have a lot of undesirable side effects, including gastrointestinal disorders that might result in stomach ulcers [41]. The main goal of this research was to develop novel anti-inflammatory drug candidates. The anti-inflammatory effects of all the compounds were assessed in vitro using the method of inhibition of albumin denaturation, as well as the ex vivo immunohistochemical method.

**Figure 3:**
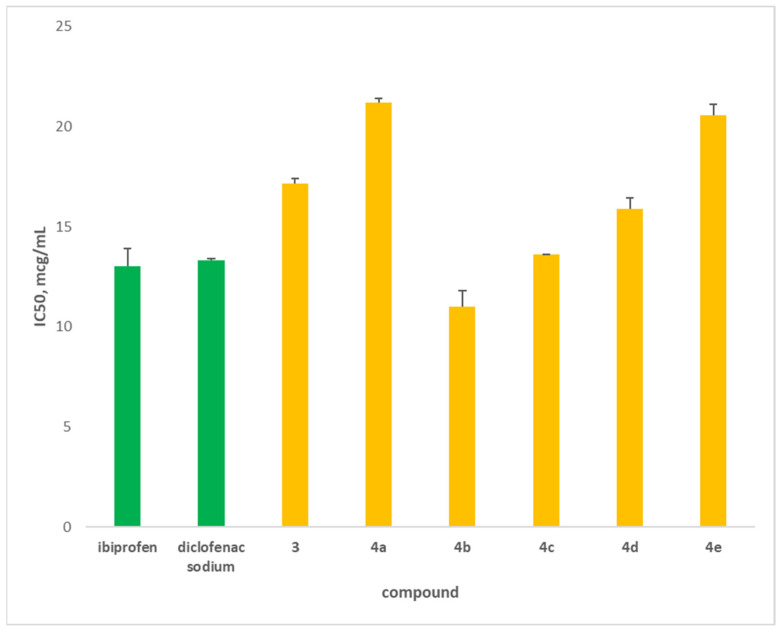
Inhibition of albumin denaturation (in μg/mL) of newly synthesized hybrids (yellow). Diclofenac and ibuprofen (green) were used as standards.

In vitro, analysis of anti-inflammatory activity was assessed as the inhibition of albumin denaturation, estimating the degree of denaturation resistance of the albumin molecule. Anti-denaturation of the human albumin method was used to evaluate the anti-inflammatory properties of the novel hybrids (Figure 3).

The novel hybrids were compared to two well-known anti-inflammatory drugs, diclofenac and ibuprofen. The main purpose of this study was to evaluate the prevention of albumin denaturation for novel hybrids. This approach estimates the degree of denaturation resistance of the albumin molecule. The data showed that hybrid **4b** (11 μg/mL) possessed the highest degree of albumin protection against denaturation compared to the standards (Figure 3). The hybrid **4c** (13.6 μg/mL) has similar protection as that of diclofenac and ibuprofen, followed by **4d** (15.9 μg/mL). The other hybrids have lower-than-standard activity. We can explain these data with the structure of the hybrids, which have phenyl, substituted phenyl or benzyl substituents.

Based on the obtained data, we can conclude that three of the novel molecules exerted a very good anti-inflammatory effect in preventing albumin denaturation. To confirm these results, additional ex vivo tests were conducted, evaluating effect of the hybrids on Interleukin-1 expression.

##### Ex Vivo Immunohistochemical Analysis

In terms of general pathology, inflammation is one of the adaptive responses to a variety of injuries, including those caused by physical, chemical, and biological factors [42]. IL-1β plays a role in resolving acute inflammation, resulting in the initiation of adaptive anti-tumor responses; however, chronic inflammatory conditions increase the risk of developing cancer [43]. The cornerstone of metabolic disorders, along with the synthesis of cytokines through lipotoxicity-mediated processes, phenotypic changes in B and T cells, and macrophage infiltration into adipose tissue, is chronic and low-grade inflammation [44,45]. The pro-inflammatory cytokines IL-2, IL-8, and TNF-a are signs of intestinal imbalance. The anti-inflammatory cytokine IL-10 can inhibit the pro-inflammatory cytokines IFN-, IL-2, IL-3, and TNF, which are generated by macrophages and TH1 cells [46]. The cytokine IL-1β, on the other hand, has a direct effect on neurons and smooth muscle in the stomach [47]. IL-1β is a potent pro-inflammatory cytokine produced by cells of the innate immune system. It is produced without the use of a signal sequence and does not secrete proteins in the usual manner; instead, it uses one or more unconventional secretion pathways.

**Figure 4:**
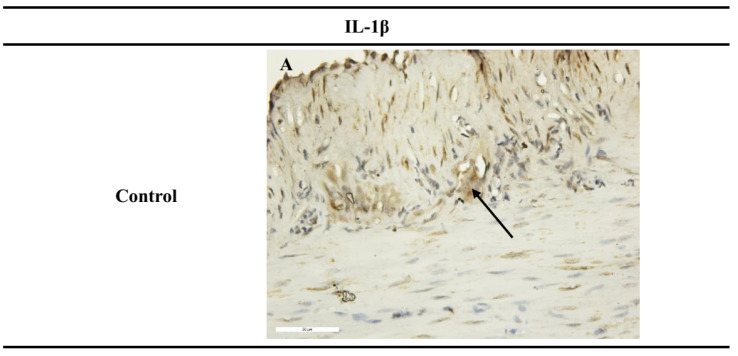

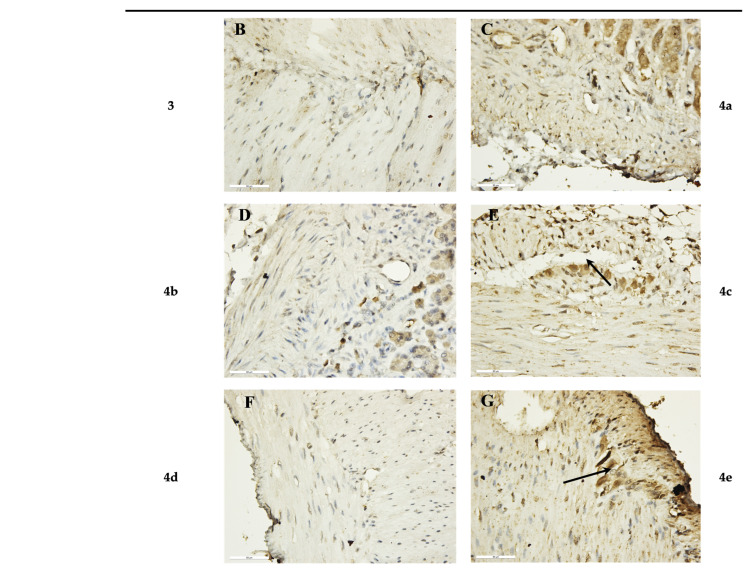
Immunohistochemical results of SM after 1 h incubation period. (**A**) Control, with weak expression of IL-1β in the myenteric plexus (black arrows) (×400); (**B**,**D**,**F**) preparations incubated with the novel hybrids **3**, **4b**, **4d**, which did not show expression for IL-1β (×400); (**C**) preparations incubated with substance **4a**, with weak expression of IL-1β (×400); (**E**,**G**) preparations incubated with hybrids **4c** and **4e**, with increased intensity and density in IL-1β expression in the myenteric plexus (black arrows) (×400).

In our experiments, a semiquantitative analysis of the immunohistological expression of IL-1β in smooth muscle preparations (SMPs) incubated with the newly synthesized hybrids was used (Figure 4).

Analysis of the IL-1β expression results in the stomach SMPs showed a higher number of IL-1β-positive cells in the section when treated with compounds **4c** and **4e** individually (Table 3). No response to IL-1β was observed in the preparations incubated with the newly synthesized compounds **3**, **4b**, and **4d**. Preparations incubated with compound **4a** showed a very weak non-specific response for IL-1β.

The results of the immunohistochemical analysis showed that substances **3**, **4b,** and **4d** have anti-inflammatory activity, as we reported a reduced-to-none expression of IL-1β receptors on SM and afferent neurons in the SMPs compared to the control ones. Based on the obtained results, we can conclude that these three hybrids **3**, **4b**, and **4d** have the potential to suppress inflammatory processes in different regions of the gastrointestinal tract [48].

In comparisons with the control data, the stained-slide semi-quantitative analysis was classified as either positive or negative. The outcomes demonstrated that exposure to either IL-1 at nanomolar concentrations enhanced neuronal excitability. Depolarization of the membrane potential, a reduction in membrane conductance, and an increase in action potential discharge made up the excitatory action. The natural human receptor antagonist for IL-1 inhibited IL-1′s excitatory activity [48].

Finally, the novel hybrids **3**, **4b**, and **4d** have an antagonistic impact in reducing the excitatory action of IL-1.

#### 2.3.2. DFT (Density Functional Theory) Calculation

**Figure 5:**
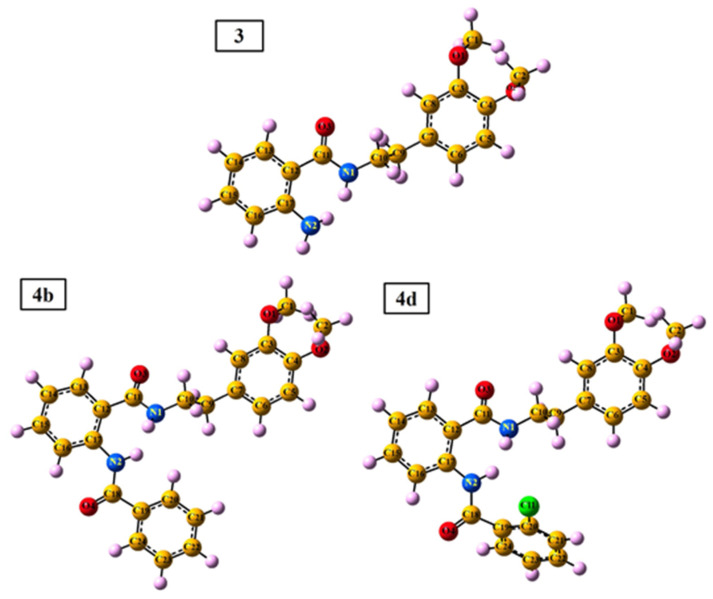
The 3D optimized geometries of the **3**, **4b**, and **4d** compounds, as well as their atom numbering.

DFT calculation was performed for all the synthesized compounds, but to avoid repetition with previously published data [20] the DFT analysis was focused on three most potent of them **3**, **4b**, and **4d**. The 3D optimized geometries of the **3**, **4b**, and **4d** compounds are demonstrated in Figure 5. The values of electronic energy for the **3**, **4b**, and **4d** compounds are found as −995.29, −1339.76, and −1799.37 Hartree, respectively.

**Figure 6:**
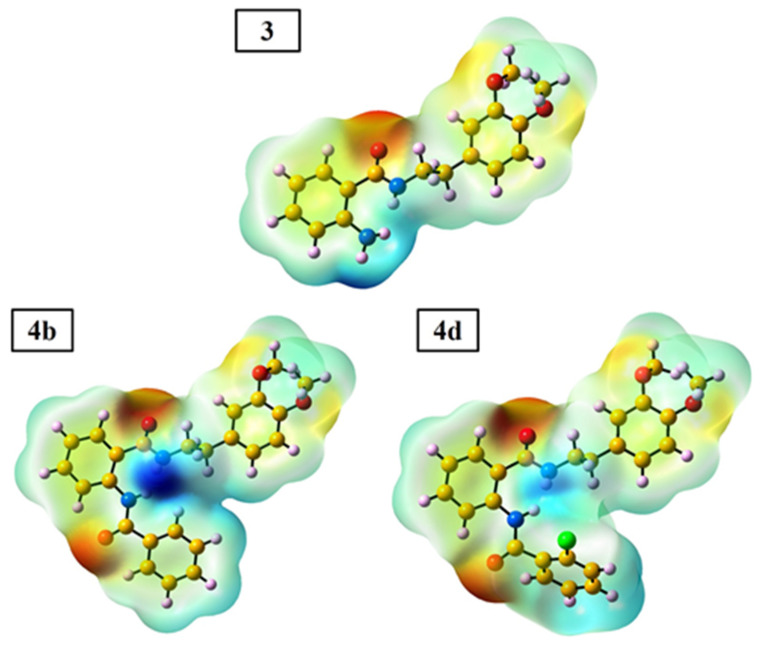
The MEP map of the **3**, **4b**, and **4d** compounds obtained from DFT calculation.

The molecular electrostatic potential (MEP) map is a crucial tool for understanding a compound’s relative polarity and the reactivity of its components [49]. The MEP surface reveals information about the compound’s site of reactivity such as electrophilic and nucleophilic sites, hydrogen bonding interactions, and charge distribution [50]. Depending on the reaction, the MEP map shows a variety of colors, including red, yellow, green, blue, and white. The blue color illustrates the positive potential area and refers to nucleophilic sites, while the red color reflects the negative potential area and is associated with electrophilic sites, and the green color indicates zero potential [51]. The MEP diagram of **3**, **4b**, and **4d** compounds is shown in Figure 6. For compound **3**, the corresponding blue area is localized on the N1H and N2H_2_ groups, while the red color is located around the C11=O3 group. Therefore, the amine groups (N1H and N2H_2_) and C11=O3 group are suitable for nucleophilic and electrophilic attack, respectively. For the **4b** and **4d** compounds, the corresponding blue area is located in the N1H and N2H groups, while the red color is located around the C11=O3 and C18=O4 groups. Therefore, the N1H and N2H groups are suitable for nucleophilic attack and the C11=O3 and C18=O4 groups are suitable for electrophilic attack.

**Figure 7:**
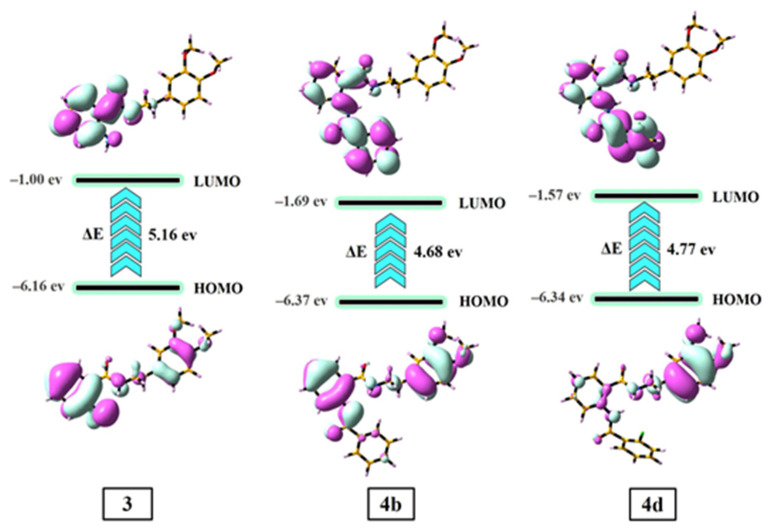
The HOMO-LUMO energy surfaces of the **3**, **4b**, and **4d** compounds.

The highest occupied molecular orbital (HOMO) and lowest unoccupied molecular orbital (LUMO) of the compounds influence their stability, chemical reactivity, and electronic behavior [52]. The HOMO-LUMO orbital of the **3**, **4b**, and **4d** compounds are depicted in Figure 7.

In compound **3**, the electron cloud of HOMO is mainly located all over the molecule, while the electron cloud of LUMO is delocalized on the benzamide group. In the **4b** and **4d** compounds, the electron cloud of HOMO is situated all over the molecule, while the electron cloud of LUMO is mainly delocalized on the benzamide and R group. The high value of the HOMO-LUMO energy gap (ΔE = E_LUMO_ − E_HOMO_) reveals the high stability and small chemical reactivity of the compounds and vice versa. The ΔE values of the **3**, **4b**, and **4d** compounds were calculated to be 5.16, 4.68, and 4.77 eV, respectively. These results indicate that **4b** and **4d** are more chemically reactive and less stable than compound **3**. The trend of increasing chemical reactivity and stability of the compounds are as follows: **4b** > **4d** > **3** and **3** > **4d** > **4b**, respectively.

Using E_HOMO_ and E_LUMO_, the quantum chemical components, i.e., chemical potential (Pi = −χ), chemical softness (σ = 1/η), chemical hardness (η = (E_LUMO_ − E_HOMO_)/2), electronegativity (χ = −(E_HOMO_ + E_LUMO_)/2), and electrophilicity index (ω = Pi^2^/2η) were calculated [53]. The data are presented in Table 4.

Hardness and softness are two vital elements in evaluating the chemical reactivity index. Soft compounds are more easily able to interact with biological molecules than hard compounds. As a result, biological activity increases as material softness rises and material hardness decreases (**4b** > **4d** > **3**) [54]. A chemical species with a higher ω value is a better electron density acceptor, whereas one with a lower value of the parameter correlates to a better capacity to supply electrons [55]. The order of the ability of the electron density acceptors from best to worst is **4b** > **4d** > **3**. The negative value of chemical potential (Pi) demonstrated evidence of the compounds’ stability. As a result, the produced compounds do not spontaneously breakdown to the original elements [56].

#### 2.3.3. Albumin Simulation

**Figure 8:**
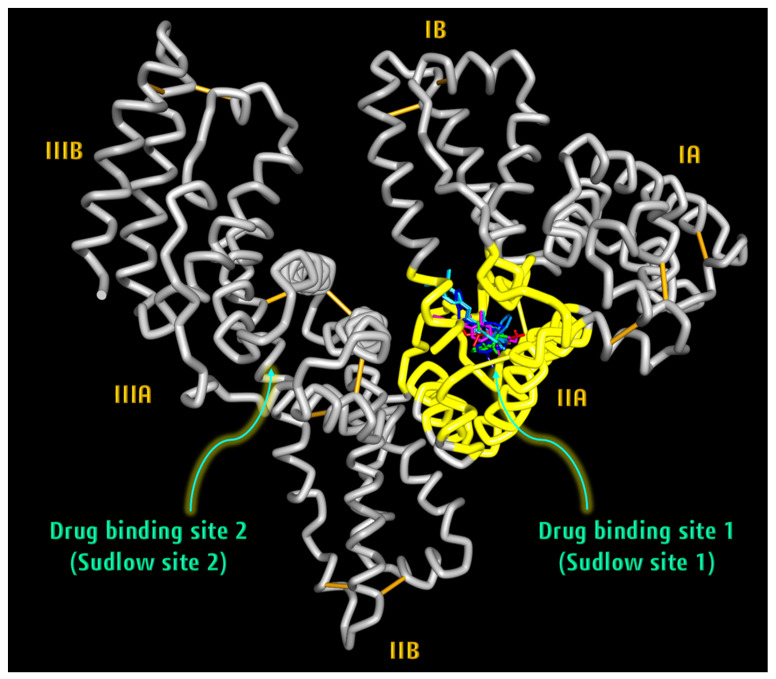
Binding pose of **3** and **4a**–**e** compounds during the interaction with HSA.

To explain better the anti-inflammatory activity results, molecular docking simulations were established. Docking simulation is a computer-based technique for finding macromolecule–small compound interactions. Non-covalent biochemical interactions between the macromolecules and the small compounds may be easily represented and evaluated using this approach [57]. Furthermore, results from in vitro anti-inflammatory activity can be examined and linked with binding affinity and interaction mode acquired from docking investigations. To determine the binding affinity and interaction mode, as well as to explain the anti-inflammatory activities, the molecular docking simulation of the **3** and **4a**–**e** compounds was investigated against human serum albumin (HSA). Since the HSA structure possesses two important drug binding sites (Sudlow sites I and II) [58], docking simulations were conducted for these sites with a grid size of 50 × 50 × 50 Å. The obtained finding for two sites demonstrated that the binding free energy value (ΔG) for the Sudlow site I is more negative than Sudlow site II, suggesting that the **3** and **4a**–**e** compounds interact with the Sudlow site I of HSA. The binding pose of synthesized compounds with HSA is illustrated in Figure 8.

**Figure 9:**
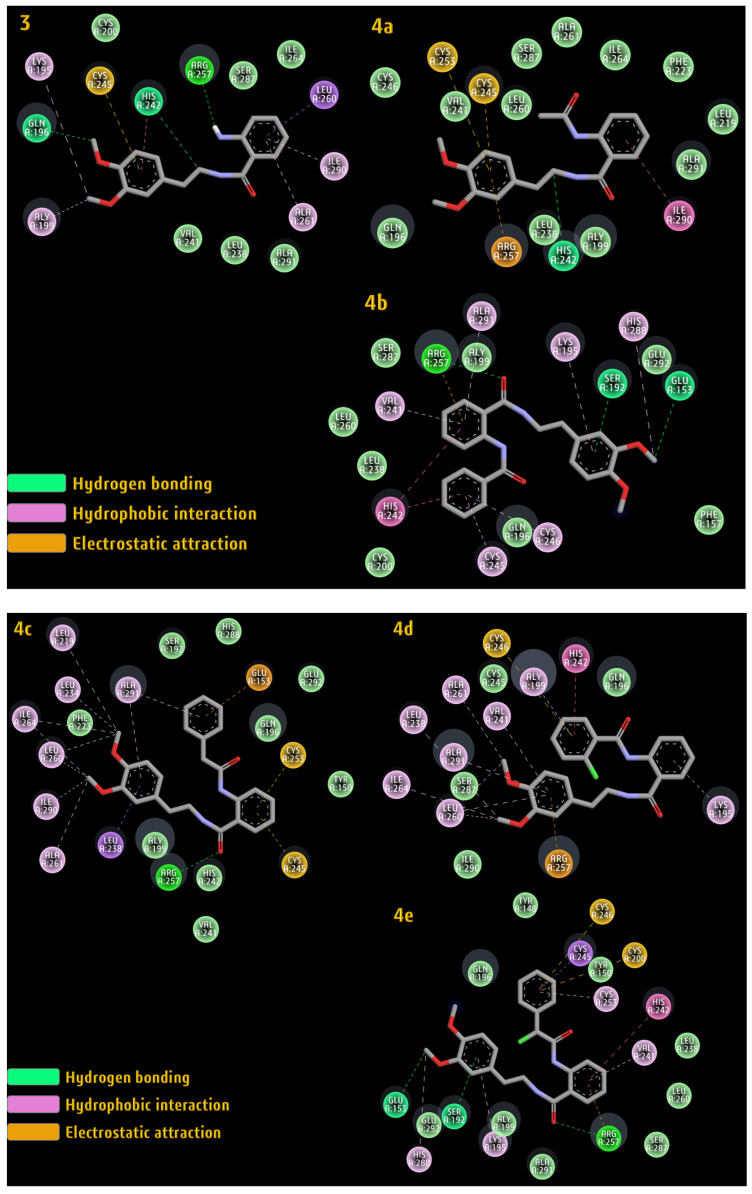
The amino acid residues of HSA are involved in the interaction with compounds **3** and **4a**–**e**.

The amino acid residues of HSA involved in the interaction with the synthesized compounds are represented in Figure 9.

The calculated ΔG value for the title compounds along with the important interaction involved between these compounds and the amino acid residue of HSA are presented in Table 5. The docking simulation indicated that the **3**, **4b**, and **4d** compounds possess higher binding affinities than the **4a**, **4c**, and **4e** compounds, which confirms the in vitro and ex vivo results. Furthermore, docking demonstrated that the nature of the binding process between the novel hybrids and HSA depends heavily on hydrogen bonding, hydrophobic interaction, and electrostatic attraction.

The results indicate that the most active compounds are **3**, **4b**, and **4d**. The large number of interactions between these compounds and amino acid residues of HSA can stabilize the albumin structure. During inflammatory processes, these interactions prevent albumin from denaturation [59,60].

An inflammatory process can present itself in a variety of ways and is the body’s natural protective response. The symptoms vary depending on which part of the body is experiencing an inflammatory process, but they are always linked to tissue destruction. The novel hybrids have a homoveratrylamine moiety; therefore, the ex vivo investigation on the spasmolytic effect was conducted for complete evaluation of their drug properties.

#### 2.3.4. Ex Vivo Smooth Muscle-Relaxant Activity

Antispasmodics are used to treat irritable bowel syndrome (IBS) and functional gastrointestinal disorders that cause abdominal pain and changes in bowel function. Anticholinergics and direct SM relaxants are the two categories of antispasmodics used in IBS. Anticholinergics are a subclass of antispasmodic drugs that work by inhibiting the action of the endogenous neurotransmitter acetylcholine (ACh) on the autonomic nervous system, which is responsible for involuntary processes. Anticholinergics block the cell signals that cause smooth muscle in the digestive and urinary tracts to contract [61].

In comparison to conventional anticholinergics, a a family of direct smooth muscle relaxants, such as Cimetropium, Mebeverin, Ottilonium bromide, Pinaverium bromide, and Trimebutin are more successful at treating IBS, and have fewer adverse effects. These drugs function by exchanging sodium and calcium transport [62]. Direct relaxants are exclusively used to treat IBS and intestinal spasms because they only affect the smooth muscle of the digestive tract by interfering with its spontaneous contractile activity. In our previous research, we showed the effect of mebeverine precursors on isolated SMPs. The focus of the current work is on the synthesis and biological evaluation of novel anthranilic acid hybrids as potential drug candidates against inflammatory diseases including gastrointestinal.

Gastrointestinal SM has been used to assess the direct or indirect influence of many new drugs, substances, and molecules. We previously reported investigations of ex vivo contractions in rat gastric smooth muscles (SM model) after the influence of isoquinolines on the intracellular Ca^2+^ concentration [33]. SM contractions can be divided into two types: phasic and tonic. The mechanisms and signal pathways that cause phasic and tonic contractions vary greatly among smooth muscles in different tissues and species [63]. In SM from the stomach and other gastrointestinal parts, ACh and its analogs like carbachol (CCh) produce contractions by activating muscarinic receptors. It is generally assumed that the muscarinic M2 and M3 receptors (M2:M3 = 3:1–5:1) play a key role in mediating this activity [64]. CCh is a choline carbamate and a positively charged quaternary ammonium compound. It is a strong promoter of interstitial cells of Cajal (ICC) activity, which is mediated through the calcium-activated chloride channel. CCh in a concentration of 10^−7^ mol/L was added to provoke high contractile tension and the newly synthesized compounds were added to the organ bath [65]. We found that the induced phasic and tonic contractions of hybrids **3** and **4a**–**e** precontracted SM tissues with 10^−7^ mol/L CCh.

In the conditions thus created in the organ bath, the phasic and tonic contractions induced by **3** and **4a**–**e** were clearly distinguished, and the contractions of SMPs in the presence of **3** (n = 6), **4a** (n = 8), **4b** (n = 6), **4c** (n = 6), **4d** (n = 6), and **4e** (n = 7) were evaluated. To analyze the differences in the phasic and tonic contractions, we obtained the cumulative concentration–response curves of the two types of contractions in the concentration range of 10^−7^ to 10^−4^ mol/L. Contractile responses induced by CCh were taken as the 100% values, and all subsequent responses were expressed as a percentage of this value.

**Figure 10:**
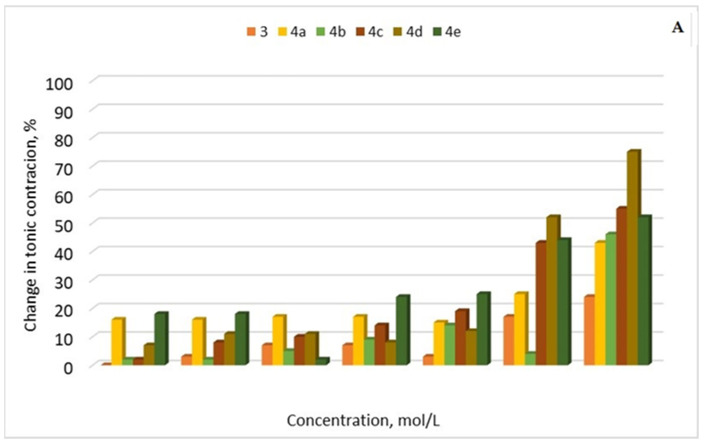

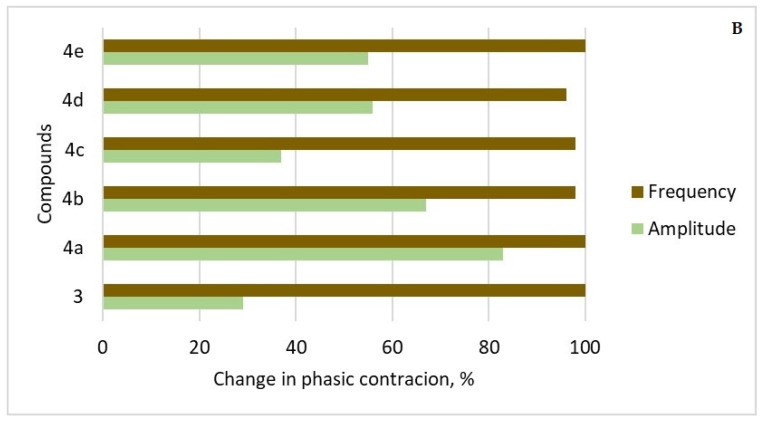
(**A**) Relaxation percentage induced by compounds **3** and **4a**–**e** (10^−7^ to 10^−4^ mol/L) on precontracted SM tissues with 10^−7^ mol/L CCh. Contractile responses induced by CCh were taken as 100%, and all subsequent responses were expressed as a percentage of this value. (**B**) Percentage changes in the amplitude and frequency of spontaneous CA elicited by compounds **3** and **4a**–**e**. (n = 8).

Three of the newly synthesized hybrids (**4c**, **4d**, and **4e**) caused a significant change in the tone of the SMP. The most pronounced relaxation reaction was observed at a concentration of 5 × 10^−5^ mol/L for all three substances, respectively: **4c**—43%, **4d**—52%, and **4e**—44% (Figure 10A). At this submaximal concentration, we assessed the change in the amplitude and frequency of phasic contractions.

After the administration of **3** and **4a**–**e** (5 × 10^−5^ mol/L), the frequency of phase contractions was between 96% to 100% for all the compounds (Figure 10B). The amplitude of the contractile response, which is the other key sign of phasic contractions, was lowered to varied degrees in the six tested drug candidates. The reduction was over 55% in the presence of **4e** and **4d**, 67% and 83% in the presence of **4b** and **4a**, respectively, and less than 37% in the presence of **3** and **4c**.

In this study, we examined the relationships between the **3** and **4a**–**e** quantity and the contractile response to clarify the regulation mechanisms of the two types of contractions. The biological activity of the novel hybrids is expressed in a significant change in tonic contractions, without affecting the amplitude and frequency of contractions. For further evaluation of the results from the animal model test, it is necessary to clarify the exact signaling pathway affected.

Finally, considering the well-known antimicrobial activity of both fragments of the novel hybrids, the thorough biological evaluation of the compounds was conducted, assessing their antimicrobial potential.

#### 2.3.5. Antimicrobial Activity

Reduced microbiota diversity brought on by gut microbiota disruption affects the production of short-chain fatty acids and antimicrobial metabolites. Patients who suffer from irritable bowel syndrome (IBS) often have bacterial overgrowth in the small intestine [66]. Anthranilic acid amides are known as very good antimicrobials [67]. 2-phenylethylamines, on the other hand, exerted modest antimicrobial activity against *Staphylococcus aureus* and *Bacillus subtilis* [68], strong activity against Gram-positive *Bacillus subtilis*, *Mycobacterium smegmatis*, *Listeria monocytogenes*, and *Staphylococcus aureus* and moderate activity against *Candida albicans* [69], as well as against *Listeria monocytogenes* [70].

In our experiments, six Gram-positive bacteria, six Gram-negative bacteria, two yeasts, and six fungi were used. The hybrid molecule and its diamides were tested in vitro for their antimicrobial activity against human pathogenic bacteria and economically relevant phytopathogenic fungi. The inhibition zones of bacterial and fungal growth caused by the novel compounds are outlined in Table 6. The obtained results were compared to previously synthesized hybrids [20] as the positive control. The methanol used as a solvent for the samples did not show any antimicrobial effect.

We observed that only the hybrids **3**, **4b**, and **4d** exerted modest antimicrobial activity against Gram-positive *Micrococcus luteus*, and Gram-negative bacteria, including the most pathogenic *Klebsiella pneumoniae, Escherichia coli*, and *Pseudomonas aeruginosa,* as well as *Penicillium chrysogenum,* whereas diamides **4a**, **4c**, and **4e** did not show any antimicrobial activity. We can explain this with the chemical structure of the compounds. Those with the benzene ring **4b** or substituted with chlorine benzene ring **4d** showed higher activity. Despite this, the newly synthesized compounds showed lower antimicrobial activity than previously described hybrids [20]. We can explain this results with the substitution with methoxy groups instead of chlorine in the novel hybrids.

## 3. Materials and Methods

### 3.1. Chemicals

All solvents and reagents were purchased from Merck (Merck KGaA, Darmstadt, Germany). Ethidium bromide (EB), DAPI (4′,6-diamidino-2-phenylindole), highly polymerized CT-DNA (type 1), and Tris–HCl buffer were obtained from Aldrich company. Melting points were determined using a Boetius hot stage apparatus and were uncorrected. All the compounds were characterized using ^1^H-NMR, ^13^C-NMR, IR, and HRESIMS. The purity of these compounds was determined by TLC using several solvent systems of different polarity. TLC was carried out on precoated 0.2 mm Fluka silica gel 60 plates (Merck KGaA, Darmstadt, Germany), using chloroform:diethyl ether:n-hexane = 6:3:1 as a chromatographic system. Neutral Al_2_O_3_ was used for column chromatographic separation. The products, after evaporation of the solvent, were purified using recrystallization from diethyl ether. IR spectra were determined on a VERTEX 70 FT-IR spectrometer (Bruker Optics, Ettlingen, Germany). Spectra of ^1^H-NMR and ^13^C-NMR were recorded on a Bruker Avance III HD 500 spectrometer (Bruker, Billerica, MA, USA) at 500 MHz (^1^H-NMR) and 125 MHz (^13^C-NMR), respectively. Chemical shifts are given in relative ppm and were referenced to tetramethylsilane (TMS) (δ = 0.00 ppm) as an internal standard; the coupling constants are indicated in Hz. The NMR spectra were recorded at room temperature (ac. 295 K). HRESIMS spectra were acquired in positive mode on Q Exactive Plus (ThermoFisher Scientific, Inc., Bremen, Germany) mass spectrometer, equipped with a heated HESI-II source. Operating conditions for the HESI source used in a positive ionization mode were +3.5 kV spray voltage, 320 °C capillary and probe heater temperature, a sheath gas flow rate of 36 a.u., an auxiliary gas flow rate of 11 a.u., a spare gas flow rate of 1 a.u. [a.u. refer to arbitrary values set by the Exactive Tune software 2.4] and an S-Lens RF level of 50.00. Nitrogen was used for sample nebulization and collision gas in the HCD cell. The aliquots of 1 µL of the solutions of the samples (ca. 20 µg mL^−1^) were introduced into the mass spectrometer via LC system Thermo Scientific Dionex Ultimate 3000 RSLC (Thermo Fisher Scientific, Germering, Germany) consisting of a 6-channel degasser SRD-3600, high-pressure gradient pump HPG-3400RS, autosampler WPS-3000TRS, and column compartment TCC-3000RS equipped with narrow bore Hypersil GOLD™ C18 (2.1 × 50 mm, 1.9 μm) column. Each chromatographic run was carried out isocratically with a mobile phase consisting of water/acetonitrile/methanol/acetic acid (25:50:25:0.2). The solvent flow rate was 300 μL min^−1^. Full MS—SIM was used as an MS experiment in negative and positive mode, where the resolution, automatic gain control (AGC) target, maximum injection time (IT), and mass range were 70,000 (at *m/z* 200), 3 × 10^6^, 100 ms, and *m/z* 100–500, respectively. Xcalibur (Thermo Fisher Scientific, Waltham, MA, USA) ver. 4.0 was used for data acquisition and processing.

### 3.2. Synthetic Methods Experimental Protocols and Spectral Data

#### 3.2.1. Synthesis of Hybrid Molecule 2-Amino-N-(3-chlorophenethyl)benzamide **3**

A mixture of isatoic anhydride (1.63 g, 10 mmol) and 2-(3-chlorophenyl)ethylamine (2.10 mL, 15 mmol) in dichloromethane (30 mL) was stirred overnight at rt. The resulting solution was filtered on the neutral Al_2_O_3_ and concentrated. Spectral data confirmed the structure of the hybrid molecule 3 (Supplementary Materials Figures S1–S4).

2-amino-N-(3,4-dimethoxyphenethyl)benzamide (**3**): ^1^H NMR (500 MHz, CDCl_3_) δ 2.88 (t, J = 6.8 Hz, 2H), 3.66 (dd, J = 10, 5 Hz, 1H), 3.86 (s, 3H, OCH_3_), 3.88 (s, 3H, OCH_3_), 5.45 (broad s,1H, NH_2_), 6.16 (s, 1H, NH), 6.65–6.62 (m, 1H), 6.71 (dd, J = 8.6, 1.0, 1H), 6.80–6.76 (m, 2H), 6.84 (d, J = 8.1, 1H), 7.21 (dd, J = 11.3, 4.4, 2H); ^13^C NMR (126 MHz, CDCl_3_) δ 169.23, 149.07, 148.21, 147.73, 132.27, 131.48, 127.00, 120.69, 117.53, 116.93, 116.45, 111.99, 111.45, 55.94, 55.85, 40.94, 35.25; FT-IR, cm^−1^: 3417 νas (-N-H, -NH_2_), 3328 νs (-N-H, -NH_2_), ν (-N-H, >NH-amide), 2997 νas (Csp^3^-H, -OCH_3_), 2962, 2932 νas (Csp^3^-H, -NH-CH_2_), 2906 νs (Csp^3^-H, -OCH_3_), 2832 νs (Csp^3^-H, -NH-CH_2_), 1634 ν (>C=O), secondary amide I, 1583 ν (C-C=C, -Ph1,2,4), 1562 ν (C-C=C, -Ph_ortho_), ν (C-C=C, -Ph1,2,4), δ (-NH), 1516 δ (N-H), ν (C-N), trans-secondary amide II, 1447 δas (-NH-CH_2_-), 1437 δs (-CH_2_-), 1375 δs(-OCH_3_), 1294 ν (C-N), secondary amide III, 1263 δ (-Csp^2^-H, -Ph_ortho_), 1158 δ (-Csp^2^-H, -Ph_ortho_), ρ (-CH_2_-)/ν(N-C) in –NH-CH_2_, 1140 δ (-Csp^2^-H, -Ph_ortho_), ρ (-CH_2_-)/ν(N-C) in –NH-CH_2_, 1026 δ (-Csp^2^-H, -Ph_ortho_), ν (N-C)/ρ(-CH_2_-) in -NH-CH_2_, 991 γ(Csp^2^-H, -Ph_ortho_); HRMS Electrospray ionization (ESI) *m/z* calcd. for [M + H]^+^ C_17_H_21_O_3_N_2_^+^ = 301.15467, found 301.15428 (mass error ∆m = −1.29 ppm)

#### 3.2.2. Diamides Synthesis **4a**–**e**; Typical Procedure

To a solution of 3 mmol 2-amino-N-(3-chlorophenethyl)benzamide **3**, 3.5 mmol of the corresponding acyl chloride in dichloromethane (10 mL) was added. Then, 3.4 mmol N(C_2_H_5_)_3_ was added in 10 min. In about 30 min, the reaction mixture was washed consequently with diluted HCl (1:4), Na_2_CO_3_, and H_2_O, then dried with anhydrous Na_2_SO_4_, filtered on the short column filled with neutral Al_2_O_3_, and concentrated.

2-acetamido-N-(3,4-dimethoxyphenethyl)benzamide (**4a**): ^1^H-NMR (500 MHz, CDCl_3_) δ 2.19 (s, 3H, COCH_3_), 2.9 (t, J = 6.8, 2H, CH_2_), 3.68 (q, J = 5, 10, 2H, CH_2_), 3.86 (s, 3H, OCH_3_), 3.87 (s, 3H, OCH_3_), 6.51 (t, J = 5.1, 1H, CONH), 6.76-6.85 (m, 3H, Ar), 7.01 (td, J = 1, 7.6, 1H, Ar), 7.32 (dd, J = 1.5, 7.8, 1H, Ar), 7.43 (td, J = 1.7, 7.9, 1H, Ar), 8.54 (d, J = 7.8, 1H, Ar), 11.05 (broad s, 1H, CONH); ^13^C-NMR (126 MHz, CDCl_3_) δ 169.1, 168.97, 149.1, 147.9, 139.5, 132.5, 131.05, 126.39, 122.7, 121.46, 120.7, 120.42, 111.9, 111.48, 55.94, 55.88, 41.2, 35.07, 25.3; FT-IR, cm^−1^: 3308 ν (-N-H, >NH-amide), 2993 νas (Csp^3^-H, -OCH_3_), 2957 νas (Csp^3^-H, -NH-CH_2_), 2834 νs (Csp^3^-H, -NH-CH_2_), 1681, 1639 ν(>C=O), secondary amide I, 1599, 1588, 1464, 1444 ν(C-C=C, -Ph_ortho_), 1302, 1279 v(C-N), secondary amide III, 1137 ρ(-CH2-)/v(N-C) in –NH-CH_2_, 1036 δ(-Csp^2^-H, -Ph_ortho_), ν(N-C)/ρ(-CH_2_-) in –NH-CH_2_; HRMS Electrospray ionization (ESI) *m/z* calcd for [M + H]^+^ C_19_H_23_O_4_N_2_^+^ = 343.16523, found 343.16473 (mass error ∆m = −1.47 ppm).

2-benzamido-N-(3,4-dimethoxyphenethyl)benzamide (**4b**): ^1^H-NMR (500 MHz, CDCl_3_) δ 2.92 (t, J = 6.8, 2H, CH_2_), 3.72 (q, J = 5, 10, 2H, CH_2_), 3.85 (s, 3H, OCH_3_), 3.87 (s, 3H, OCH_3_), 6.53 (t, J = 5.4, 1H, CONH), 6.76–6.85 (m, 2H, Ar), 7.04 (td, J = 1.5, 7.6, 1H, Ar), 7.37 (dd, J = 1.5, 7.8, 1H, Ar), 7.47–7.59 (m, 4H, Ar), 8.05-8.06 (m, 1H, Ar), 8.11–8.19 (m, 1H, Ar), 8.78 (dd, J = 1, 8.3, 1H, Ar), 12.13 (broad s, 1H, CONH); ^13^C-NMR (126 MHz, CDCl_3_) δ 169.2, 165.65, 1149.16, 147.68, 139.85, 134.8, 132.65, 131.89, 131.04, 130.59, 130.14, 128.91, 128.8, 128.47, 127.4, 126.47, 122.89, 121.6, 120.7, 111.9, 11.48, 55.94, 55.88, 41.23, 35.04; FT-IR, cm^−1^: 3374 ν(-N-H, >NH-amide), 3312 ν(-N-H, >NH-amide), 2991 νas (Csp^3^-H, -OCH_3_), 2963 νas (Csp^3^-H, -OCH_3_), 2935 νas (Csp^3^-H, >CH_2_), 2840 νs (Csp^3^-H, >CH_2_), 1683 ν (>C=O), secondary amide I, 1623 ν (>C=O), secondary amide I, 1592 ν (C-C=C, -Ph_ortho_), 1516 ν (C-C=C, -Ph_ortho_), ν (C-C=C, -Ph1,2,4), δ (N-H) and ν (C-N), trans-secondary amide II, 1312 δ (N-H) and ν (C-N), trans-secondary amide II, 1296 ν (C-N), secondary amide III, 951, 932 γ (-Csp^2^-H, -Ph_mono_), γ (-Csp^2^-H, -Ph1,2,4), 896 γ (-Csp^2^-H, -Ph_mono_), γ (-Csp^2^-H, -Ph_ortho_), γ (-Csp^2^-H, -Ph1,2,4); HRMS Electrospray ionization (ESI) *m/z* calcd for [M + H]^+^ C_24_H_25_O_4_N_2_^+^ = 405.18088, found 405.18019 (mass error ∆m = −1.71 ppm).

N-(3,4-dimethoxyphenethyl)-2-(2-phenylacetamido)benzamide (**4c**): ^1^H-NMR (500 MHz, CDCl_3_) δ 2.85 (t, J = 6.8, 2H, CH_2_), 3.61-3.64 (m, 2H, CH_2_), 3.73 (s, 2H, CH_2_C_6_H_5_), 3.85 (s, 3H, OCH_3_), 3.88 (s, 3H, OCH_3_), 6.37 (t, J = 5.4, 1H, CONH), 6.73-6.77 (m, 2H, Ar), 6.84 (d, J = 8.3, 1H, Ar), 6.98 (td, J = 1.2, 7.7, 1H, Ar), 7.27–7.29 (m, 2H, Ar), 7.37–7.43 (m, 5H, Ar), 8.55 (dd, J = 1, 8.3, 1H, Ar), 11.09 (broad s, 1H, CONH); ^13^C-NMR (126 MHz, CDCl_3_) δ 169.9, 168.8, 149.14, 147.85, 139.27, 134.7, 132.38, 131.12, 129.45, 129.0, 128.8, 127.2, 126.3, 122.86, 121.49, 120.7, 111.96, 111.48, 55.95, 55.87, 45.65, 41.28, 34.99; FT-IR, cm^−1^: 3326 ν (-N-H, >NH-amide), 3258 ν (-N-H, >NH-amide), 2991 νas (Csp^3^-H, -OCH_3_), 2948 νas (Csp^3^-H, >CH_2_), 2920 νas (Csp^3^-H, >CH_2_), 2868 νs (Csp^3^-H, -OCH_3_), 2837 νs (Csp^3^-H, >CH_2_), 1654 ν (>C=O), secondary amide I, 1634 ν (>C=O), secondary amide I, 1605 ν (C-C=C, -Ph_ortho_), ν (C-C=C, -Ph1,2,4), ν (C-C=C, -Ph_mono_), 1512 ν (C-C=C, -Ph_ortho_), ν (C-C=C, -Ph1,2,4), δ (N-H) and ν (C-N), trans-secondary amide II, 1311 δ (N-H) and ν (C-N), trans-secondary amide II, 1136 δ(-Csp^2^-H, -Ph_ortho_), δ(-Csp^2^-H, -Ph1,2,4), ρ(-CH_2_-)/ν(N-C) in –NH-CH_2_, 1044 δ(-Csp^2^-H, -Ph_ortho_), δ(-Csp^2^-H, -Ph1,2,4), ρ(-CH_2_-)/ν(N-C) in –NH-CH_2_, 1028 δ(-Csp^2^-H, -Ph_mono_), 936 γ(-Csp^2^-H, -Ph_ortho_), γ(-Csp^2^-H, -Ph1,2,4); HRMS Electrospray ionization (ESI) *m/z* calcd for [M + H]^+^ C_25_H_27_O_4_N_2_^+^ = 419.19653, found 419.19579 (mass error ∆m = −1.77 ppm).

2-chloro-N-(2-((3,4-dimethoxyphenethyl)carbamoyl)phenyl)benzamide (**4d**): ^1^H-NMR (500 MHz, CDCl_3_) δ 2.86 (t, J = 6.8, 2H, CH_2_), 3.64 (q, J = 5, 10, 2H, CH_2_), 3.83 (s, 3H, OCH_3_), 3.86 (s, 3H, OCH_3_), 6.62 (broad s, 1H, CONH), 6.73–6.83 (m, 2H, Ar), 6.81–6.83 (m, 1H, Ar), 7.07 (td, J = 1, 7.6, 1H, Ar), 7.33–7.41 (m, 3H, Ar), 7.45–7.52 (m, 2H, Ar), 7.65 (dd, J = 7.3, 2, 1H, Ar), 8.72 (d, J = 8.3, 1H, Ar), 11.53 (broad s, 1H, CONH); ^13^C-NMR (126 MHz, CDCl_3_) δ 168.85, 165.57, 149.1, 147.8, 138.95, 136.0, 134.4, 133.26, 132.58, 132.25, 131.35, 131.05, 130.62, 127.18, 126.6, 123.5, 121.86, 120.7, 111.9, 111.48, 55.94, 55.87, 41.25, 34.98; FT-IR, cm^−1^: 3354 ν (-N-H, >NH-amide), 2958 νas (Csp^3^-H, -OCH_3_), 2934 νas (Csp^3^-H, >CH_2_), 2913 νas (Csp^3^-H, >CH_2_), 2836 νs (Csp^3^-H, >CH_2_), 1668 ν (>C=O), secondary amide I, 1641 ν (>C=O), secondary amide I, 1592 ν (C-C=C, -Ph_ortho_), ν (C-C=C, -Ph1,2,4), 1543 δ (N-H) and ν (C-N), trans-secondary amide II, 1512 ν (C-C=C, -Ph_ortho_), ν (C-C=C, -Ph1,2,4), δ (N-H) and ν (C-N), trans-secondary amide II, 1310 δ (N-H) and ν (C-N), trans-secondary amide II, 1287 ν (C-N), secondary amide III, 1155, 1139 δ (-Csp^2^-H,-Ph_ortho_), ρ (-CH2-)/v (N-C) in –NH-CH_2_, 1027 δ (ortho-Ph-Cl), 900 γ (-Csp^2^-H, -Ph_ortho_), γ (-Csp^2^-H, -Ph1,2,4); HRMS Electrospray ionization (ESI) *m/z* calcd for [M + H]^+^ C_24_H_24_O_4_ClN_2_^+^ = 439.14191, found 439.14124 (mass error ∆m = −1.53 ppm).

2-(2-chloro-2-phenylacetamido)-N-(3,4-dimethoxyphenethyl)benzamide (**4e**): ^1^H-NMR (500 MHz, CDCl_3_) δ 2.91 (t, J = 6.8, 2H, CH_2_), 3.71–3.73 (m, 2H, CH_2_), 3.85 (s, 3H, OCH_3_), 3.89 (s, 3H, OCH_3_), 5.47 (s, 1H, CHCl), 6.35 (t, J = 4.9, 1H, CONH), 6.73–6.86 (m, 3H, CH(Cl) overlapped with Ar), 7.07 (td, J = 7.6; 1.5, 1H, Ar), 7.33-7.42 (m, 5H, Ar), 7.43-7.47 (m, 1H, Ar), 7.59–7.61 (m, 2H, Ar), 8.54 (dd, J = 8.3, 1, 1H, Ar), 12.05 (broad s, 1H, CONH); ^13^C-NMR (126 MHz, CDCl_3_) δ 168.67, 166.6, 149.16, 147.88, 138.65, 132.5, 129.09, 128.9, 127.89, 126.37, 123.66, 121.39, 120.7, 111.9, 111.49, 62.2, 55.97, 55.88, 41.3, 34.99; FT-IR, cm^−1^: 3321 ν(-N-H, >NH-amide), 2990 νas (Csp^3^-H, -OCH_3_), 2943 νas (Csp^3^-H, >CH_2_), 2838 νs (Csp^3^-H, >CH_2_), 1676 ν (>C=O), secondary amide I, 1624 ν (>C=O), secondary amide I, 1596 ν (C-C=C, -Ph_ortho_), ν (C-C=C, -Ph1,2,4), ν (C-C=C, -Ph_mono_), 1550 δ (N-H) and ν (C-N), trans-secondary amide II, 1513 ν (C-C=C, -Ph_ortho_), ν (C-C=C, -Ph1,2,4), δ (N-H) and ν (C-N), trans-secondary amide II, 1326 ν (C-C=C, -Ph_ortho_), 1312 δ (N-H) and ν (C-N), trans-secondary amide II, 1294 ν (C-C=C, -Ph_mono_), ν (C-N), secondary amide III, 1159 δ (-Csp^2^-H, -Ph_ortho_), δ (C-C=C, -Ph_mono_), ρ (-CH_2_-)/ν (N-C) in –NH-CH_2_, 1141 ρ (-CH_2_-)/ν(N-C) in –NH-CH_2_, 1037 ρ (-CH_2_-)/ν (N-C) in –NH-CH_2_, 1023 δ (-Csp^2^-H, -Ph_mono_), ν (C-Cl), 970 ν (-Csp^2^-H, -Ph_mono_), 957 γ (-Csp^2^-H, -Ph_mono_), 909 γ (-Csp^2^-H, -Ph_mono_), γ (-Csp^2^-H, -Ph_ortho_), γ (-Csp^2^-H, -Ph1,2,4); HRMS Electrospray ionization (ESI) *m/z* calcd for [M + H]^+^ C_25_H_26_O_4_ClN_2_^+^ = 453.15756, found 453.15694 (mass error ∆m = −1.37 ppm).

### 3.3. In Silico Predictions

#### 3.3.1. PASS Online Predictions

A computer-based program, PASS online (Prediction of Activity Spectra for Substances), was used to screen the biological activity of the compounds. The program predicts several thousand different biological activities based on the structural formula of a drug-like organic compound [34]. PASS has been used by many scientists for the discovery of new pharmaceutical agents in different therapeutic fields [35,36].

#### 3.3.2. Theoretical Prediction of Pharmacokinetic Parameters (ADME)

Physicochemical properties, drug likeness, and pharmacokinetic parameters such as ADME (absorption, distribution, metabolism, and elimination) of the synthesized prodrugs were analyzed using free SwissADME web tools. It provides a predictive model for the pharmacokinetic profiling of a drug-like compound [62].

#### 3.3.3. Theoretical Prediction of Toxicity

For predicting acute as well as organ toxicity of the compounds, the ProToxII free web tool was used. It predicts various toxicity endpoints, including acute toxicity and organ toxicities such as hepatotoxicity, cytotoxicity, carcinogenicity, mutagenicity, immunotoxicity, and toxicity targets. Toxicity class and LD50 values were also estimated [71,72].

### 3.4. DFT Calculations

The structures of the investigated compounds were optimized via a B3LYP/6-311G(d,p) level, as applied in the Gaussian 09W software [73]. The frequency calculations were conducted for each compound to verify that the structures had undoubtedly been minimized. The GaussView 6 package was used to draw the primary structures and visually display the results. The computational optimization was performed in the gas phase at the ground state. In this study, DFT calculation was employed for determining the 3D optimized structure of the compounds, as well as to analyze the molecular electrostatic potential (MEP) surface and the HOMO-LUMO diagram.

### 3.5. Molecular Docking Simulation

Docking simulation is a crucial tool for investigating the interaction of synthesized compounds with protein, which is highly helpful for drug design and development. The docking simulation was performed using the Auto Dock 4.2 [74] and Auto Dock Tools 1.5.6 [75] packages to reveal the kind of binding affinity and modes between the produced compounds and human serum albumin (HSA), as well as investigation of the anti-inflammatory activities. The crystal structure of HSA (ID: 2I2Z [76]) was extracted from the Protein Data Bank and the structure of the synthesized compounds was obtained from DFT calculation. For the docking technique, the 3D grid size of 50 × 50 × 50 Å was selected for both drug binding sites of HSA, with a specification of 0.375. The remaining options were set similarly to the previous work [20]. The Discovery Studio software v.21.1.0.20298 was used to depict docked positions and the interaction diagram.

### 3.6. Microbiological Tests

#### 3.6.1. Tested Microorganisms

Twenty tested microorganisms including six Gram-positive bacteria (*Bacillus subtilis* ATCC 6633, *Bacillus amyloliquefaciens* 4BCL-YT, *Staphylococcus aureus* ATCC 25923, *Listeria monocytogenes* NBIMCC 8632, *Listeria innocua* ATCC 33090, *Enterococcus faecalis* ATCC 19433, *Enterococcusfaecium* ATCC 19434, *Micrococcus luteus* 2YC-Y), six Gram-negative bacteria (*Salmonella enteritidis* ATCC 13076, *Klebsiella* sp.—clinical isolate, Escherichia coli ATCC 25922, *Proteus vulgaris* ATCC 6380, *Pseudomonas aeruginosa* ATCC 9027), two yeasts (*Candida albicans* NBIMCC 74, *Saccharomyces cerevisiae* ATCC 9763), and six fungi (*Aspergillus niger* ATCC 1015, *Aspergillus flavus*, *Penicillium* sp., *Rhizopus* sp., *Mucor* sp.—plant isolates, Fusarium moniliforme ATCC 38932) from the collection of the Department of Microbiology at the University of Food Technologies—Plovdiv, Bulgaria, were selected for the antimicrobial activity test.

#### 3.6.2. Culture Media

Luria–Bertani agar (LBG agar) medium supplemented with glucose was prepared according to the manufacturer’s (Laboratorios Conda S.A., Madrid, Spain) instructions: 50 g of LBG-solid substance mixture (containing 10 g tryptone, 5 g yeast extract, 10 g NaCl, 10 g glucose, and 15 g agar) was dissolved in 1 L of deionized water (pH 7.5), and then the medium was autoclaved at 121 °C for 20 min.

##### Malt Extract Agar (MEA)

MEA was prepared according to the manufacturer’s (Laboratorios Conda S.A., Madrid, Spain) instructions: 50 g of the MEA-solid substance mixture (containing 30 g malt extract, 5 g mycological peptone, and 15 g agar) was dissolved in 1 L of deionized water (pH 5.4), and then the medium was autoclaved at 115 °C for 15 min.

#### 3.6.3. Antimicrobial Activity Assay

The antimicrobial activity of the samples was determined using the agar well diffusion method. The tested bacteria *B. subtilis* and *B. amyloliquefaciens* were cultured on LBG agar at 30 °C. The test bacteria *S. aureus*, *L. monocytogenes*, *E. faecalis*, *S. enteritidis*, *Klebsiella* sp., *E. coli*, *P. vulgaris*, and *P. aeruginosa* were cultured on LBG agar at 37 °C for 24 h. The yeast C. albicans was cultured on MEA at 37 °C, while S. cerevisiae was cultured on MEA at 30 °C for 24 h. The fungi *A. niger*, *A. flavus*, *Penicillium* sp., *Rhizopus* sp., *Mucor* sp., and *F. moniliforme* were grown on MEA at 30 °C for 7 days or until sporulation.

The inocula of the tested bacteria/yeasts was prepared by homogenization of a small amount of biomass in 5 mL of sterile 0.5% NaCl. The inocula of tested fungi were prepared by the addition of 5 mL of sterile 0.5% NaCl into the tubes. After stirring by vortex V-1 plus (Biosan), they were filtered and replaced in other tubes before use. The number of viable cells and fungal spores was determined using a bacterial counting chamber Thoma (Poly-Optik, GmbH, Bad Blankenburg, Germany). Their final concentrations were adjusted to 108 cfu/mL for bacterial/yeast cells and 105 cfu/mL for fungal spores and then inoculated in LBG/MEA agar media preliminarily melted and tempered at 45–48 °C. Next, the inoculated media were transferred in a quantity of 18 mL in sterile Petri plates (d = 90 mm) (Gosselin™, Hazebrouck, France) and allowed to harden. Then, six wells (d = 6 mm) per plate were cut, and triplicates of 60 μL of each extract were pipetted into the agar wells. The Petri plates were incubated in identical conditions.

The antimicrobial activity was determined by measuring the diameter of the inhibition zones around the wells on the 24th and 48th hour of incubation. Tested microorganisms with inhibition zones of 18 mm or more were considered sensitive; moderately sensitive were those in which the zones were from 12 to 18 mm; and resistant were those in which the inhibition zones were up to 12 mm or completely missing [77].

### 3.7. Inhibition of Albumin Denaturation

Anti-denaturation assay was conducted as described by Kumari et al. [20,59,78] with slight modifications. The reaction mixture consisted of 0.5 mL of 5% aqueous solution of human albumin (Albunorm 20, Octapharma (IP) SPRL, 1070 Anderlecht, Belgium) and 0.2 mL of the tested compound, dissolved in DMSO, at different concentrations (20–500 μg/mL). The samples were incubated at 37 °C for 15 min. After that, 2.5 mL phosphate-buffered saline (pH 6.3) was added to each tube and the samples were heated for 30 min to 80 °C and then cooled for 5 min. Turbidity was measured spectrophotometrically at 660 nm (Cary 60 UV-Vis, Agilent Technologies, Santa Clara, CA 95051, USA). For the blank sample, a mixture of 2.5 mL buffer and 0.2 mL DMSO was used instead of the compounds, while the product control test lacked the compounds’ concentration having 0.5 mL serum albumin, and 2.5 mL buffer only. The percentage inhibition of protein denaturation was calculated as follows:

Percentage of inhibition denaturation = (Absorbance_control_ − Absorbance_sample_)/Absorbance_control_ × 100

The control represents 100% protein denaturation. Commercially available anti-inflammatory drugs diclofenac and ibuprofen were used for comparison. Their anti-inflammatory effect was determined as a percentage of inhibition of albumin denaturation, following the same protocol as for the novel compounds.

### 3.8. Immunohistochemical Methods

#### 3.8.1. Histology

After dissection, the SM strips were incubated for 20 min in the tissue bath using the same physiological conditions in which the CA recording experiments were carried out. Then, each SM strip was fixed in 10% neutral buffer formalin for 24–48 h and submitted for standard processing with hematoxylin and eosin staining.

The SM fragments from a rat’s gastric wall were fixed in a 10% neutral formalin solution and embedded in paraffin. Paraffin sections of 5 μm thickness were subjected to hematoxylin and eosin (H-E) staining for histochemical analysis.

#### 3.8.2. Immunohistochemistry

Immunohistochemical staining was performed on formalin-fixed, paraffin-embedded 4 μm sections after retrieval of antigenic epitopes with citrate pH 6.0, endogenous biotin, and peroxidase block. Stomach preparations were tested immunohistochemically with primary antibody IL-1β (E-AB-52153), (Elabscience Biotechnology Inc., Houston, TX, USA). Immunohistochemical (IHC) staining was performed using an Autostainer Link 48 (Dako, Santa Clara, CA, USA). Images were visualized and captured with a digital camera mounted on a Nikon Eclipse 80i microscope using NIS-Elements Advanced Research Software version 4.13 (Nikon Instruments, Tokyo, Japan).

#### 3.8.3. Analysis of Immunohistochemical Reactions

A quantitative and statistical analysis of immunohistochemical reaction using the Olympus DP-Soft image system (version 4.1 for Windows) was carried out on a Microphot-SA (Nikon, Tokyo, Japan) microscope equipped with a Camedia-5050Z digital camera (Olympus, Tokyo, Japan). The analysis was performed on sections from the SM strips from the stomach of Wistar rats (n = 6 for each group). Five sections of the SM strips were measured, and the percentage of cells expressing 5-HT_3_ in the circular and longitudinal layer of SM cells, as well as in the myenteric plexus of the stomach, was determined. Each antibody was analyzed for five fields, in each of them the average number of cells with positive unit area response at ×400 magnification.

### 3.9. Smooth Muscle Activity

#### 3.9.1. Animals, Tissues, and Preparations

Male Wistar rats with a weight of 270 ± 15 g (age 11 weeks) were used. The animals were purchased from the Animal House of Medical University Plovdiv, Bulgaria. All experimental procedures were carried out with utmost confidence following the Guidelines for the Care and Use of Laboratory Animals at the Medical University of Plovdiv, under permit No. 229/09.04.2019, Bulgaria. These procedures were approved by the current European regulations (86/609/EEC) governing the protection of animals used for experimental purposes and were executed with full confidence in strict accordance with the current Institutional Animal Care Bulgaria. Furthermore, the experimental procedures fully comply with the EU Directive 2010/63/EU.

One rat stomach was used to obtain four circular strips of muscle tissue while keeping the mucosa intact. The muscle strips had a length of 9–11 mm and a width of 1.0–1.2 mm. The number of samples is indicated by n and were obtained under conditions of continuous irrigation of tissues with pre-aerated (95% O_2_ and 5% CO_2_) Krebs solution consisting of NaCl/KCl/CaCl_2_ in a ratio 27.2/1.1/1 with a temperature of 4 °C [79].

#### 3.9.2. Measuring Smooth Muscle Tension

The corpus SMPs were prepared for the ex vivo contractile experiment. Contractions were recorded isometrically using a system of 15 mL organ baths (Tissue Organ Bath System 159920 Radnoti, Dublin, Ireland). The samples were equilibrated under a resting tension of 10 mN (1 g) in a normal physiological salt solution (Krebs solution) containing (in mM) 140.0 NaCl, 5.0 KCl, 1.2 MgCl_2_, 1.8 CaCl_2_, 23.8 NaHCO_3_, and 11.1 glucose. The pH of the solution was measured before each experiment by pH-meter HI5521 (Hanna Instruments, Smithfield, RI, USA). The bath solution was saturated with a 95% O_2_ and 5% CO_2_ mixture at 37 °C, pH 7.4. Each strip was exposed to Krebs solution until stable contractions for 40 min. All results are expressed as a percent of the maximum response in a physiological buffer (Krebs solution) [80].

#### 3.9.3. Studying the Mechanical Activity of Isolated SMPs

Experiments were conducted to investigate the dose-dependent contractile effects of compounds **3** and **4a**–**e** on stomach SMPs. For the isometrical detection of the changes in substance-evoked reactions in tissues SMPs were placed vertically in organ baths and attached by threads to a force transducer on one end and a holder for isometric tension measurement on the other end SM tissue vitality was tested by adding 10^−6^ mol/L ACh to the organ baths. The tissue was equilibrated for 60 min and every 15 min the Krebs solution was replaced. Then, an agonist was picked as a compound that would cause active contraction [81].

### 3.10. Statistical Analysis

The Instat computer program for analysis of the variance was used. The mean and standard error of each group’s mean (SEM) was calculated. A two-way ANOVA was used to compare different groups with the respective controls for repeated measurements. A *p*-value of *p* < 0.05 was considered representative of a significant difference. IBM SPSS Statistics v. 26 statistical package was used for statistical analyses.

## 4. Conclusions

In conclusion, novel hybrid diamides of isatoic anhydride with homoveratrylamine were synthesized as potential anti-inflammatory drug candidates. In silico data signified the compounds as potential orally active compounds with high gastrointestinal absorption, blood–brain barrier penetration, anti-inflammatory and spasmolytic activity, and showed no toxicity. The results showed that all the compounds exhibit a preventive effect on albumin denaturation but those having a phenyl or benzyl substituent prevented albumin’s denaturation better than common anti-inflammatory drugs diclofenac and ibuprofen. To confirm the presence of an anti-inflammatory effect in the diamides, we conducted additional ex vivo tests evaluating their effect on Interleukin-1 expression, and similar results were observed. Smooth muscle relaxant activity also verifies these data showing the hybrids to have the potential to suppress inflammatory processes in different regions of the gastrointestinal tract.

The docking analysis explained the anti-inflammatory efficacy with formation of ligand–albumin complexes with Sudlow site I, as well as stabilization of the HSA structure by forming hydrogen bonds, hydrophobic interactions, and electrostatic attractions.

The experimental results and theoretical calculations allow us to conclude that the newly synthesized hybrids present promise as excellent drug candidates, since they inherit the anti-inflammatory potential of the anthranilic acid nucleus, as well as the smooth muscle spasmolytic effect of 2-phenylethylamine residue. Future preclinical experiments will contribute to the establishment of the novel hybrids as potential drug candidates against inflammatory diseases.

## Figures and Tables

**Table 1:** Synthesis of anthranilic acid diamides.

4	R	Yield, %	mp, °C
**a**	CH_3_	80	95–97
**b**	C_6_H_5_	78	121–124
**c**	CH_2_-C_6_H_5_	79	92–93
**d**	2-Cl-C_6_H_4_	81	94–95
**e**	CH(Cl)C_6_H_5_	82	102–103

**Table 2:** Calculated ADME parameters of the compounds **3** and **4a**–**d** representing drug likeness.

Compound	MW, g/mol	XLOGP3	TPSA, Å^2^	ESOL Log S	Fraction Csp^3^	RB	BA Score	SA Score	Lipinski’s Violation
**3**	300.35	2.97	73.58	−3.51	0.24	7	0.55	2	0
**4a**	342.39	2.83	76.66	−3.51	0.26	9	0.55	2.42	0
**4b**	404.46	4.48	76.66	−4.95	0.17	10	0.55	2.73	0
**4c**	418.48	4.42	76.66	−4.82	0.20	11	0.55	2.90	0
**4d**	438.93	5.11	76.66	−5.55	0.17	10	0.55	2.81	1
**4e**	452.93	5.14	76.66	−5.58	0.20	11	0.55	3.48	0

MW: molecular weight; XLOGP3: octanol/water partition coefficient; TPSA: topological polar surface area; ESOL LogS: estimated aqueous solubility; Fraction Csp^3^: ratio of sp^3^ hybridized carbons over the total carbon count; RB: rotatable bonds; BA: bioavailability; SA: synthetic accessibility; LD_50_—predicted lethal dose to 50% of animals.

**Table 3:** Comparative measurements and expression of IL-1β and response to compounds **3** and **4a**–**e**.

Compound	Control	Response to Compound
IL-1β	++	
**3**		−
**4a**		++
**4b**		−
**4c**		+++
**4d**		−
**4e**		+++

The expression of IL-1β was characterized in a panel of SMs and neurons in the myenteric plexus. The expression level of IL-1β protein was determined by assessing the antigen expression in tissue sections using immunohistochemical staining. − absent; ++, moderate; +++, strong.

**Table 4:** The quantum chemical components of **3**, **4b**, and **4d** compounds estimated from DFT (unit: eV).

Compound	E_HOMO_	E_LUMO_	ΔE	χ	Pi	η	σ	ω
**3**	−6.16	−1.00	5.16	3.58	−3.58	2.58	0.39	2.48
**4b**	−6.37	−1.69	4.68	4.03	−4.03	2.34	0.43	3.47
**4d**	−6.34	−1.57	4.77	3.95	−3.95	2.38	0.42	3.27

**Table 5:** The values of binding free energy (ΔG) and the type of interaction between the **3** and **4a**–**e** compounds and HSA.

			Type of Interaction with Residue	
Compound	ΔG (kcal/mol)	Hydrogen Bonding	Hydrophobic Interaction	Electrostatic Attraction
**3**	−5.36	ARG257, HIS242, GLN196	LEU260, ILE290, ALA261, HIS242, LYS195, ALY199	CYS245
**4a**	−5.22	HIS242	ILE290	CYS245, CYS253, ARG257
**4b**	−6.11	ARG257, SER192, GLU153	HIS288, LYS195, ALA291, VAL241, HIS242, CYS245, CYS246	ARG257
**4c**	−4.87	ARG257	LEU238, ALA261, ILE290, LEU260, ILE264, LEU234, ALA291, LEU219	CYS245, CYS253, GLU153
**4d**	−6.21	GLN196	LYS195, HIS242, ALY199, VAL241, ALA261, ALA291, LEU238, ILE264, LEU260	CYS246, ARG257
**4e**	−5.28	ARG257, SER192, GLU153	CYS245, CYS253, HIS242, VAL241, LYS195, HIS288	CYS246, CYS200

**Table 6:** Antimicrobial activity of the tested compounds.

Inhibition Zones, mm/Anthranilic Acid Hybrid and Its Diamides			
	3	4b	4d
*Bacillus subtilis* ATCC 6633	-	-	10
*Bacillus amyloliquefaciens* 4BCL-YT	-	-	10
*Micrococcus luteus* 2YC-YT	10	-	15
*Salmonella enteritidis* ATCC 13076	10	-	-
*Salmonella typhimurium* NBIMCC 1672	8	10	8
*Klebsiella pneumoniae* ATCC 13883	-	15	-
*Escherichia coli* ATCC 25922	8	10	10
*Pseudomonas aeruginosa* ATCC 9027	8	8	10
*Aspergillus niger* ATCC 1015	12	10	8
*Penicillium chrysogenum*	10	9	8
*Rhizopus* sp.	9	-	8

dwell = 6 mm.

## Data Availability

Data is contained in the paper.

## Supplementary Materials

The following supporting information can be downloaded at: https://www.mdpi.com/article/10.3390/ph16121660/s1, Figure S1: ^1^H-NMR spectrum of compound **3**, Figure S2: ^13^C-NMR spectrum of compound **3**, Figure S3: DEPT spectrum of compound **3**, Figure S4: FT-IR spectrum of compound **3**, Figure S5: Mass spectrum of **3**, Figure S6: ^1^H-NMR spectrum of compound **4a**, Figure S7: ^13^C-NMR spectrum of compound **4a**, Figure S8: DEPT spectrum of compound **4a**, Figure S9: FT-IR spectrum of compound **4a**, Figure S10: Mass spectrum of **4a**, Figure S11: ^1^H-NMR spectrum of compound **4b**, Figure S12: ^13^C-NMR spectrum of compound **4b**, Figure S13: DEPT spectrum of compound **4b**, Figure S14: FT-IR spectrum of compound **4b**, Figure S15: Mass spectrum of **4b**, Figure S16: ^1^H-NMR spectrum of compound **4c**, Figure S17: ^13^C-NMR spectrum of compound **4c**, Figure S18: DEPT spectrum of compound **4c**, Figure S19: FT-IR spectrum of compound **4c**, Figure S20: Mass spectrum of **4c**, Figure S21: ^1^H-NMR spectrum of compound **4d**, Figure S22: ^13^C-NMR spectrum of compound **4d**, Figure S23: DEPT spectrum of compound **4d**, Figure S24: FT-IR spectrum of compound **4d**, Figure S25: Mass spectrum of **4d**, Figure S26: ^1^H-NMR spectrum of compound **4e**, Figure S27: ^13^C-NMR spectrum of compound **4e**, Figure S28: DEPT spectrum of compound **4e**, Figure S29: FT-IR spectrum of compound **4e**, Figure S30: Mass spectrum of **4e**.
