# Supplementary material for: Novel Anthranilic Acid Hybrids—An Alternative Weapon against Inflammatory Diseases
Source: Pharmaceuticals (Basel). 2023 Nov 29;16(12):1660. doi: 10.3390/ph16121660 (PMC10747134; doi:10.3390/ph16121660)

Novel anthranilic acid hybrids – an alternative weapon against inflammatory diseases

Miglena Milusheva, Mina Todorova, Vera Gledacheva, Iliyana Stefanova, Mehran Feizi-Dehnayebi, Mina Pencheva, Paraskev Nedialkov, Yulian Tumbarski, Velichka Yanakieva, Slava Tsoneva, and Stoyanka Nikolova

Table of Contents:

Figure S1: ^{1}H-NMR spectrum of compound **3**, page 2	Figure S16: ^{1}H-NMR spectrum of compound **4c**, page 17
Figure S2: ^{13}C-NMR spectrum of compound **3**, page 3	Figure S17: ^{13}C-NMR spectrum of compound **4c**, page 18
Figure S3: DEPT spectrum of compound **3**, page 4	Figure S18: DEPT spectrum of compound **4c**, page 19
Figure S4: FT-IR spectrum of compound **3**, page 5	Figure S19: FT-IR spectrum of compound **4c**, page 20
Figure S5: Mass spectrum of **3**, page 6	Figure S20: Mass spectrum of **4c**, page 21
Figure S6: ^{1}H-NMR spectrum of compound **4a**, page 7	Figure S21: ^{1}H-NMR spectrum of compound **4d**, page 22
Figure S7: ^{13}C-NMR spectrum of compound **4a**, page 8	Figure S22: ^{13}C-NMR spectrum of compound **4d**, page 23
Figure S8: DEPT spectrum of compound **4a**, page 9	Figure S23: DEPT spectrum of compound **4d**, page 24
Figure S9: FT-IR spectrum of compound **4a**, page 10	Figure S24: FT-IR spectrum of compound **4d**, page 25
Figure S10: Mass spectrum of **4a**, page 11	Figure S25: Mass spectrum of **4d**, page 26
Figure S11: ^{1}H-NMR spectrum of compound **4b**, page 12	Figure S26: ^{1}H-NMR spectrum of compound **4e**, page 27, 28
Figure S12: ^{13}C-NMR spectrum of compound **4b**, page 13	Figure S27: ^{13}C-NMR spectrum of compound **4e**, page 29
Figure S13: DEPT spectrum of compound **4b**, page 14	Figure S28: DEPT spectrum of compound **4e**, page 30
Figure S14: FT-IR spectrum of compound **4b**, page 15	Figure S29: FT-IR spectrum of compound **4e**, page 31
Figure S15: Mass spectrum of **4b**, page 16	Figure S30: Mass spectrum of **4e**, page 32

Figure S1: ^{1}H-NMR spectrum of compound 3

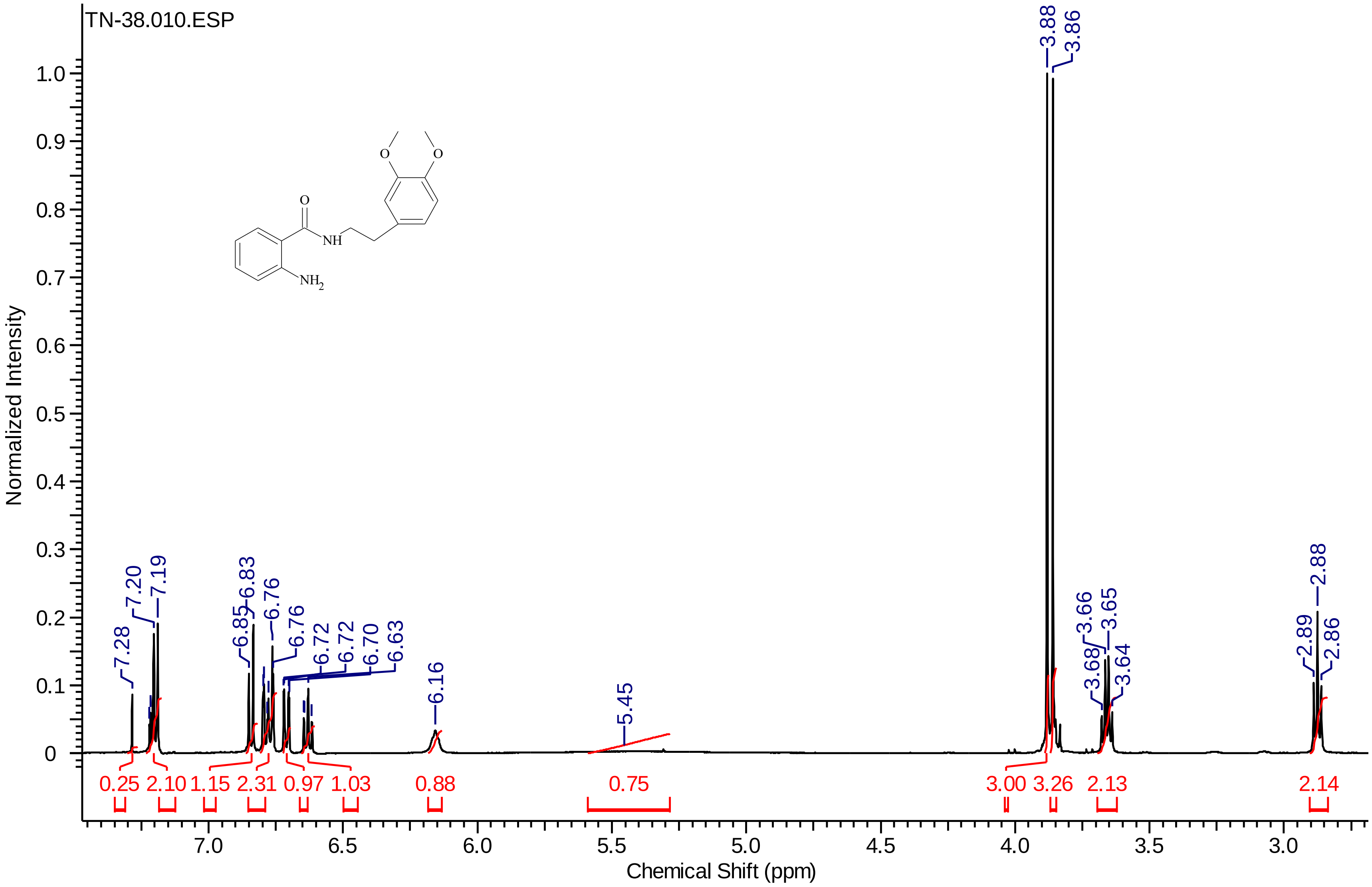

Figure S2: ^{13}C-NMR spectrum of compound **3**

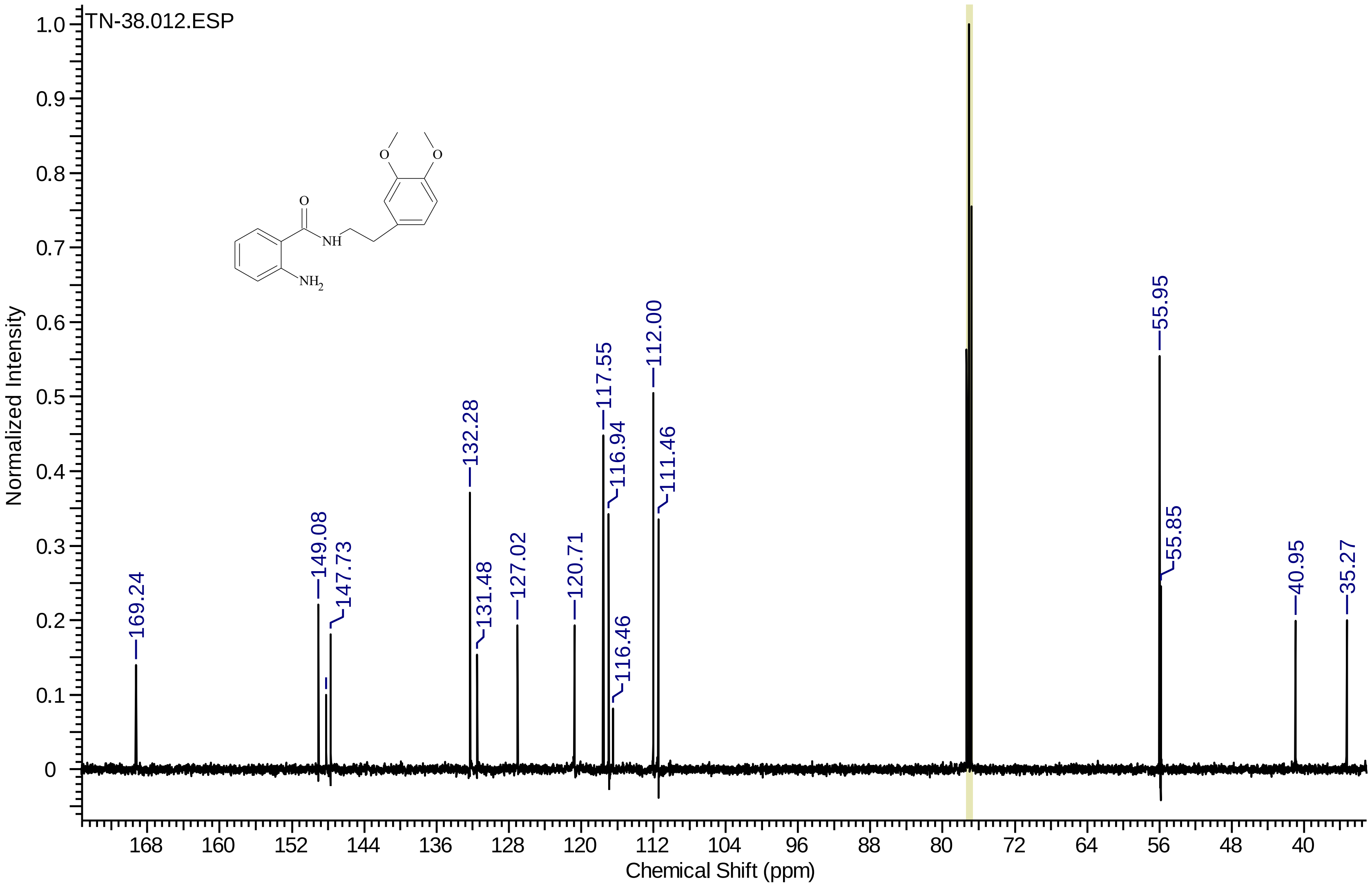

Figure S3: DEPT spectrum of compound **3**

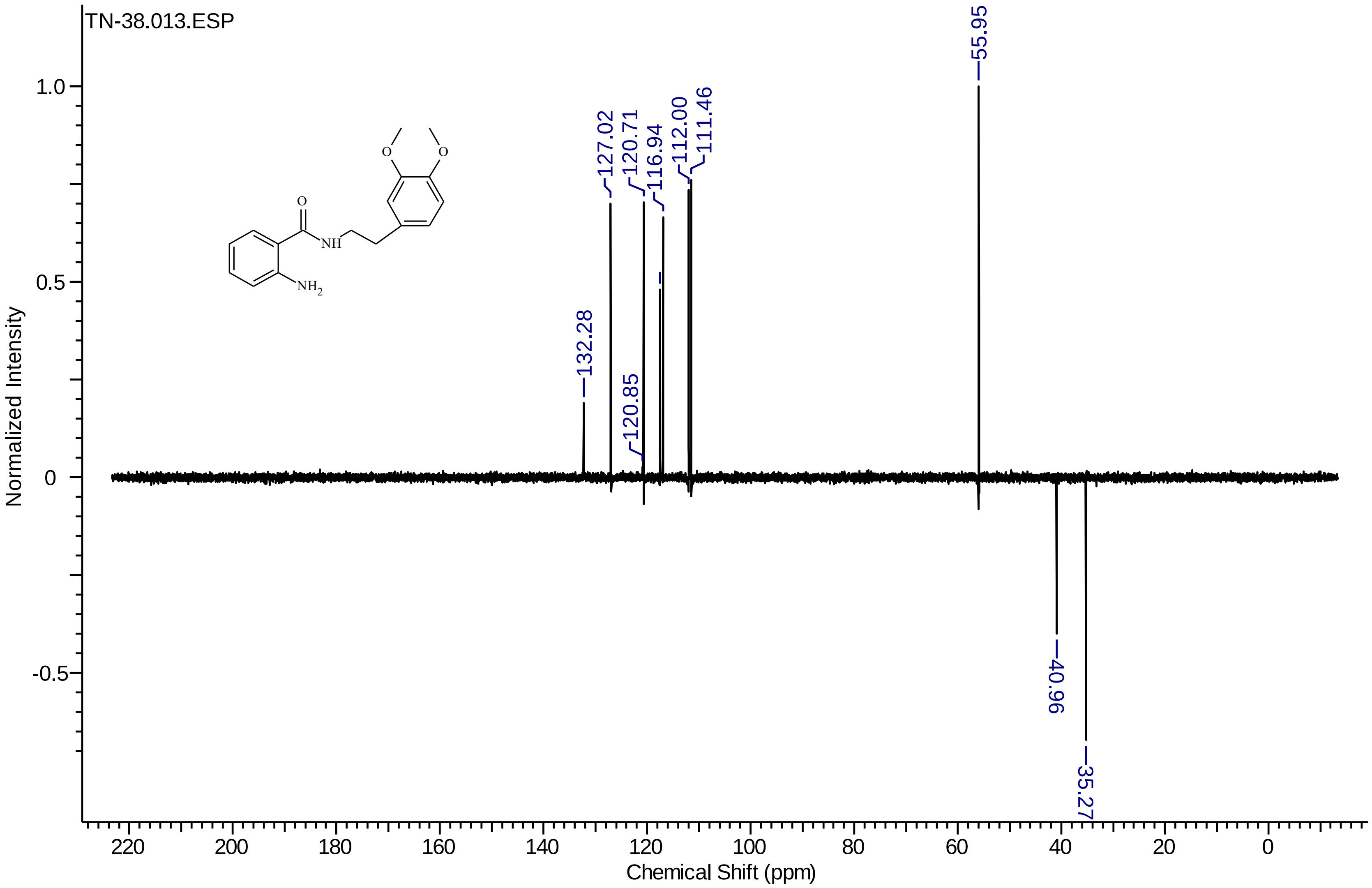

Figure S4: FT-IR spectrum of compound **3**

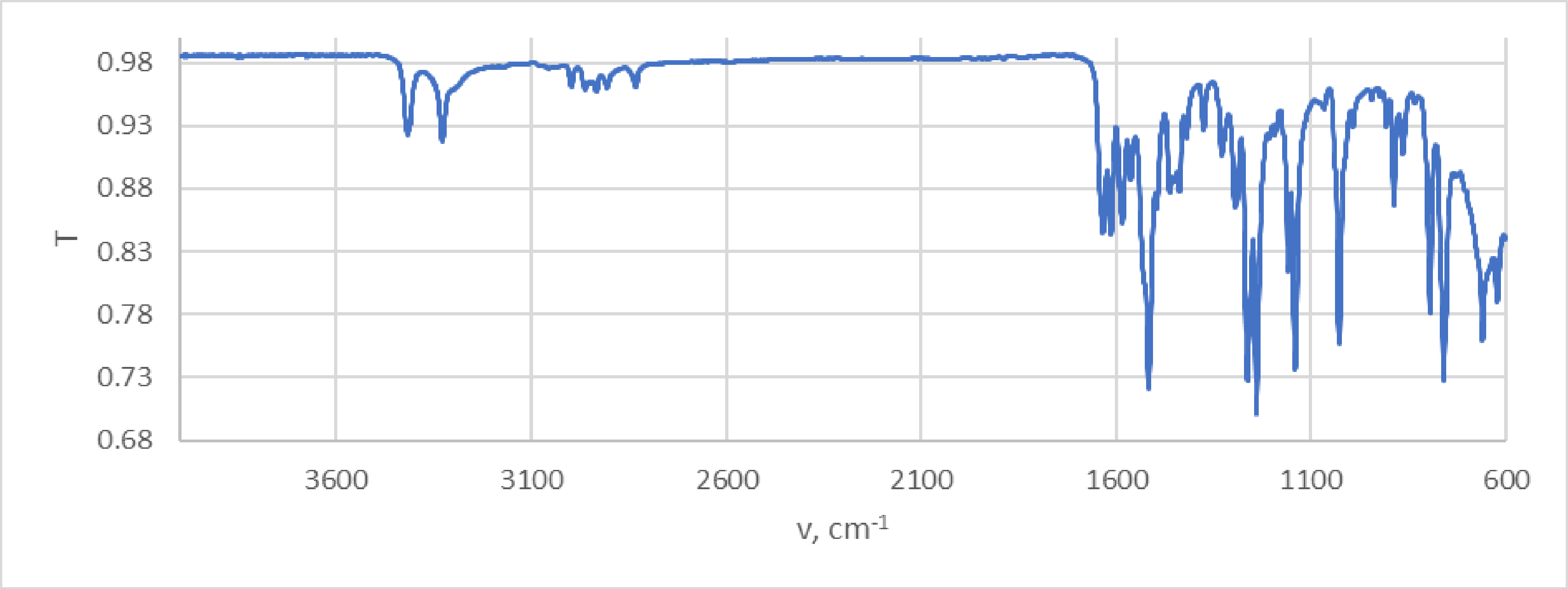

Figure S5: Mass spectrum of **3.**

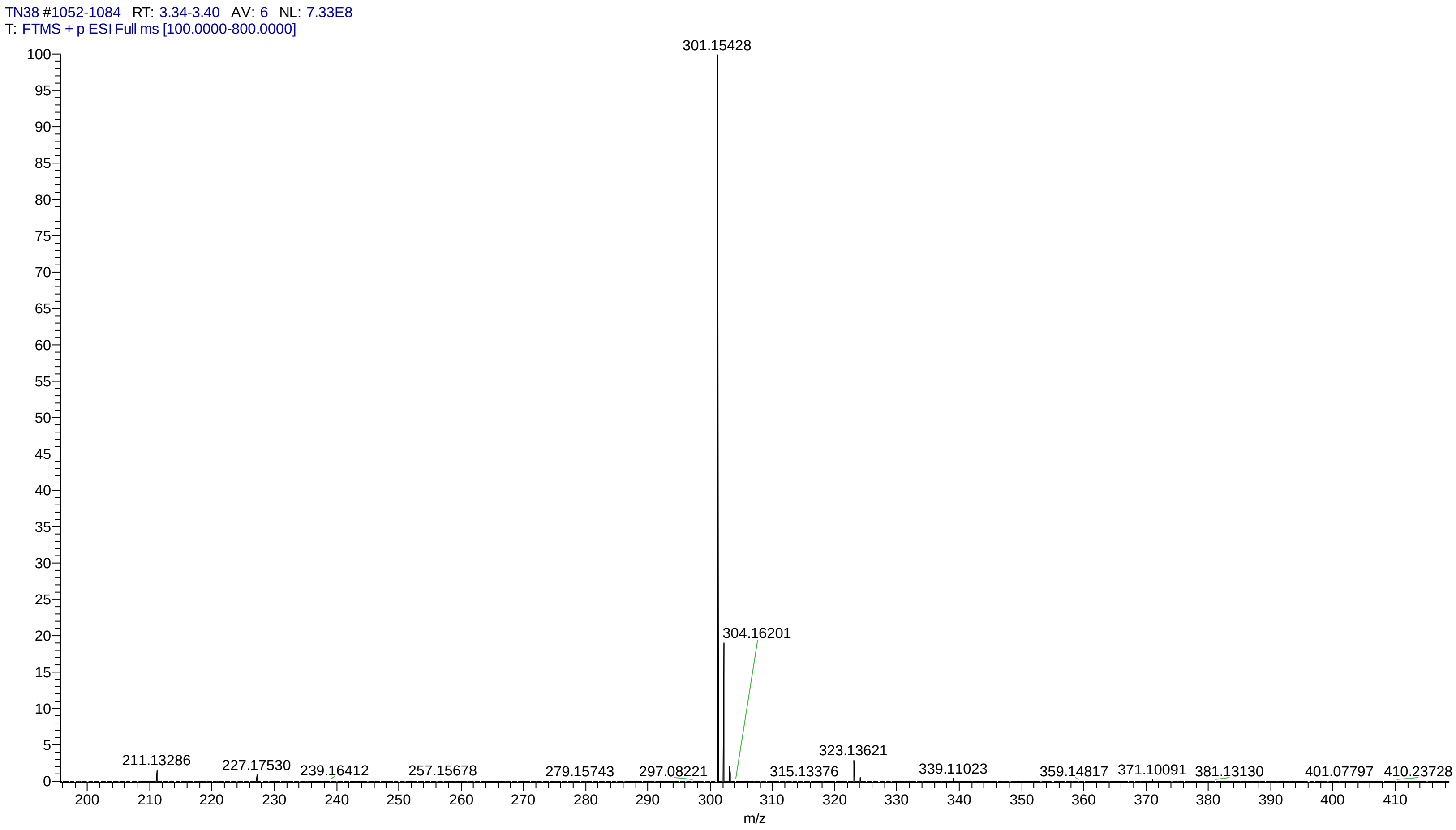

Figure S6: ^{1}H-NMR spectrum of compound **4a**

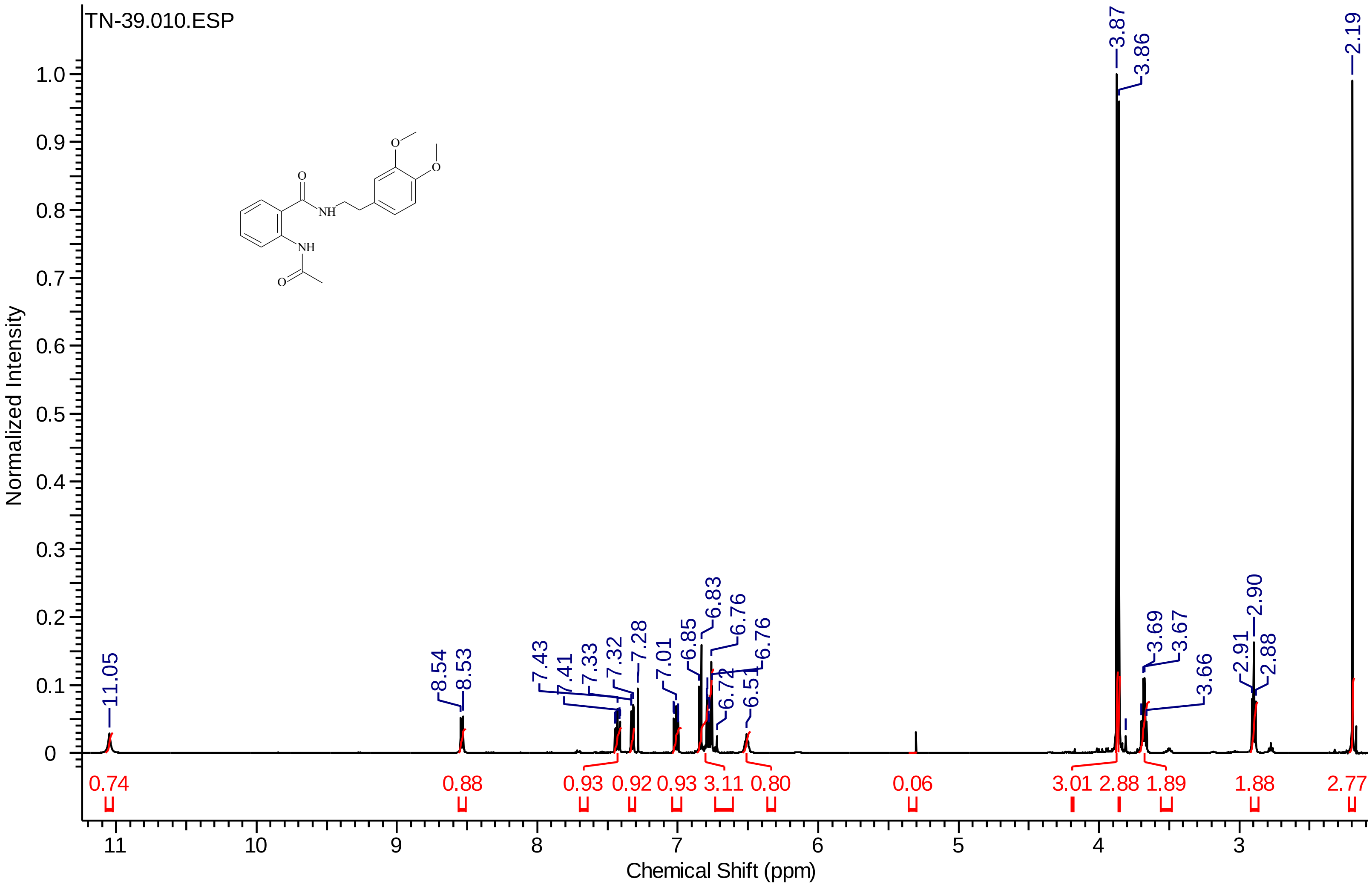

Figure S7: ^{13}C-NMR spectrum of compound **4a**

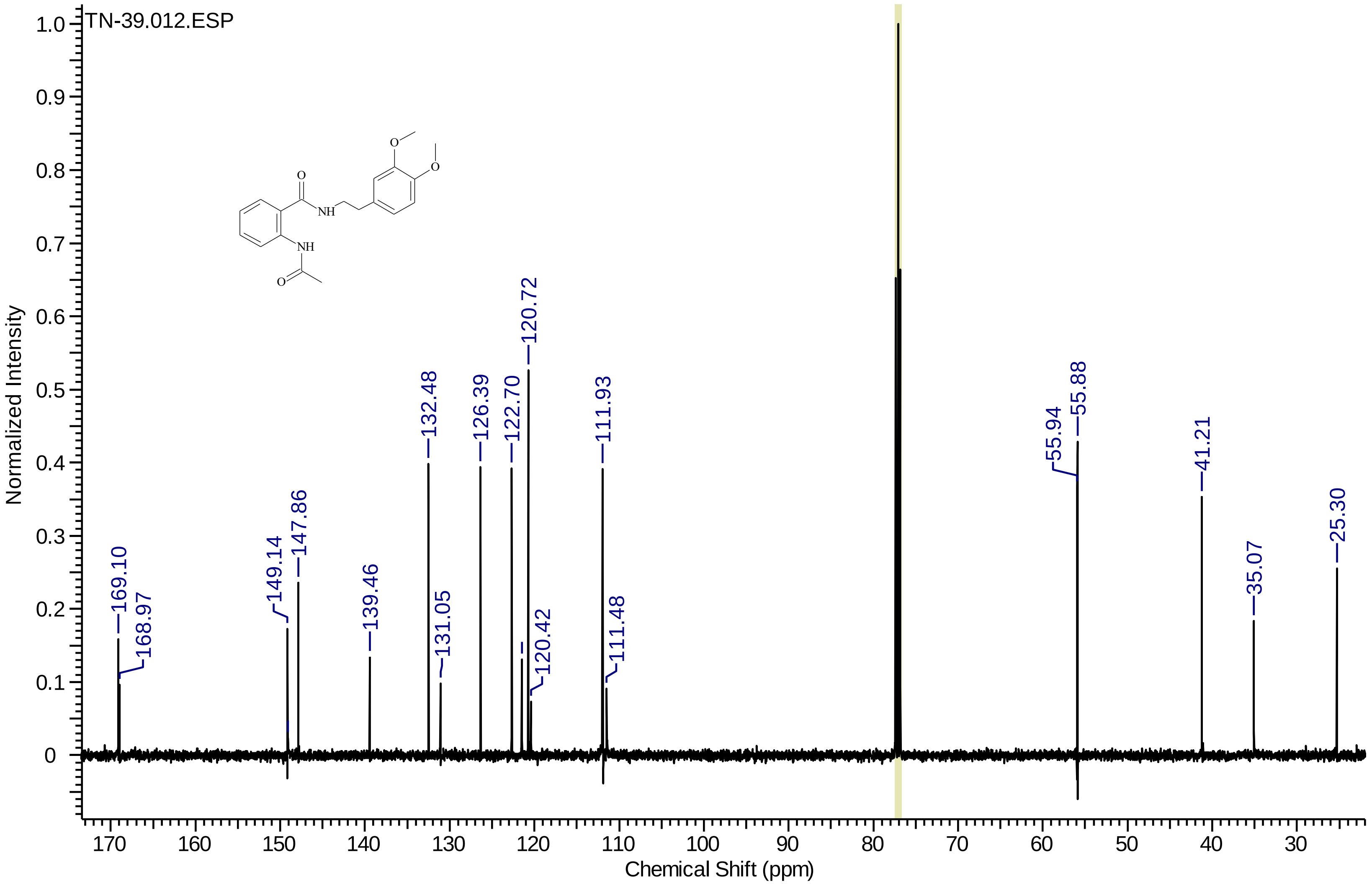

Figure S8: DEPT spectrum of compound **4a**

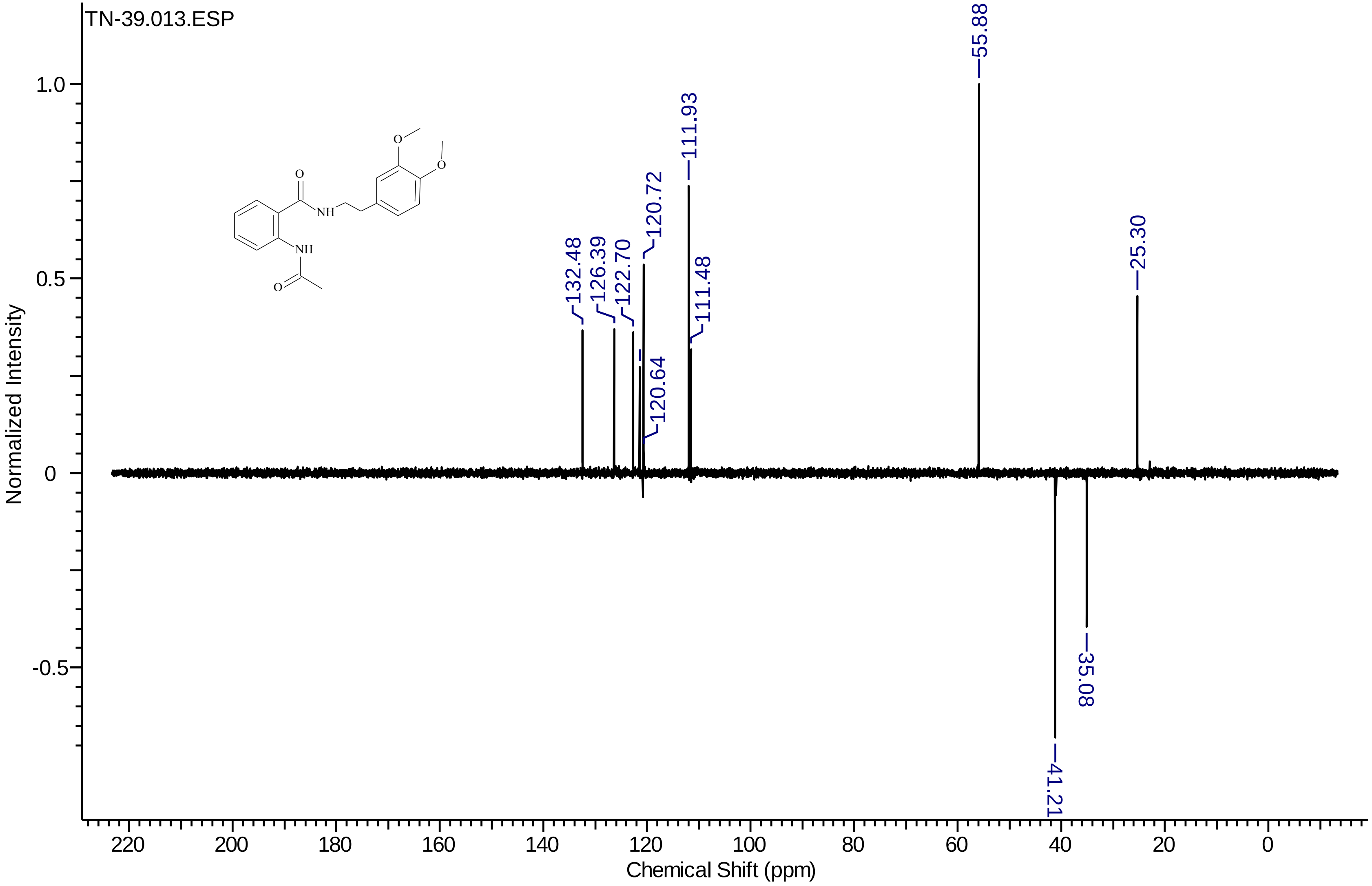

Figure S9: FT-IR spectrum of compound **4a**

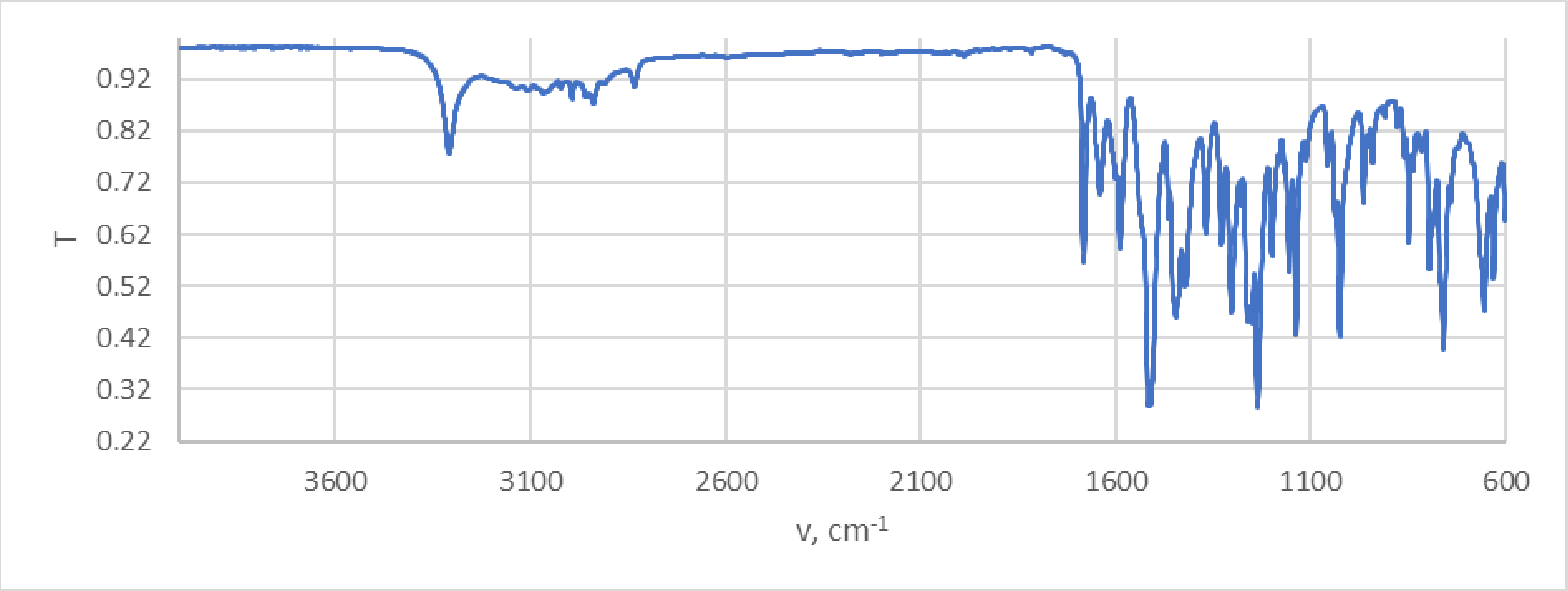

Figure S10: Mass spectrum of **4a.**

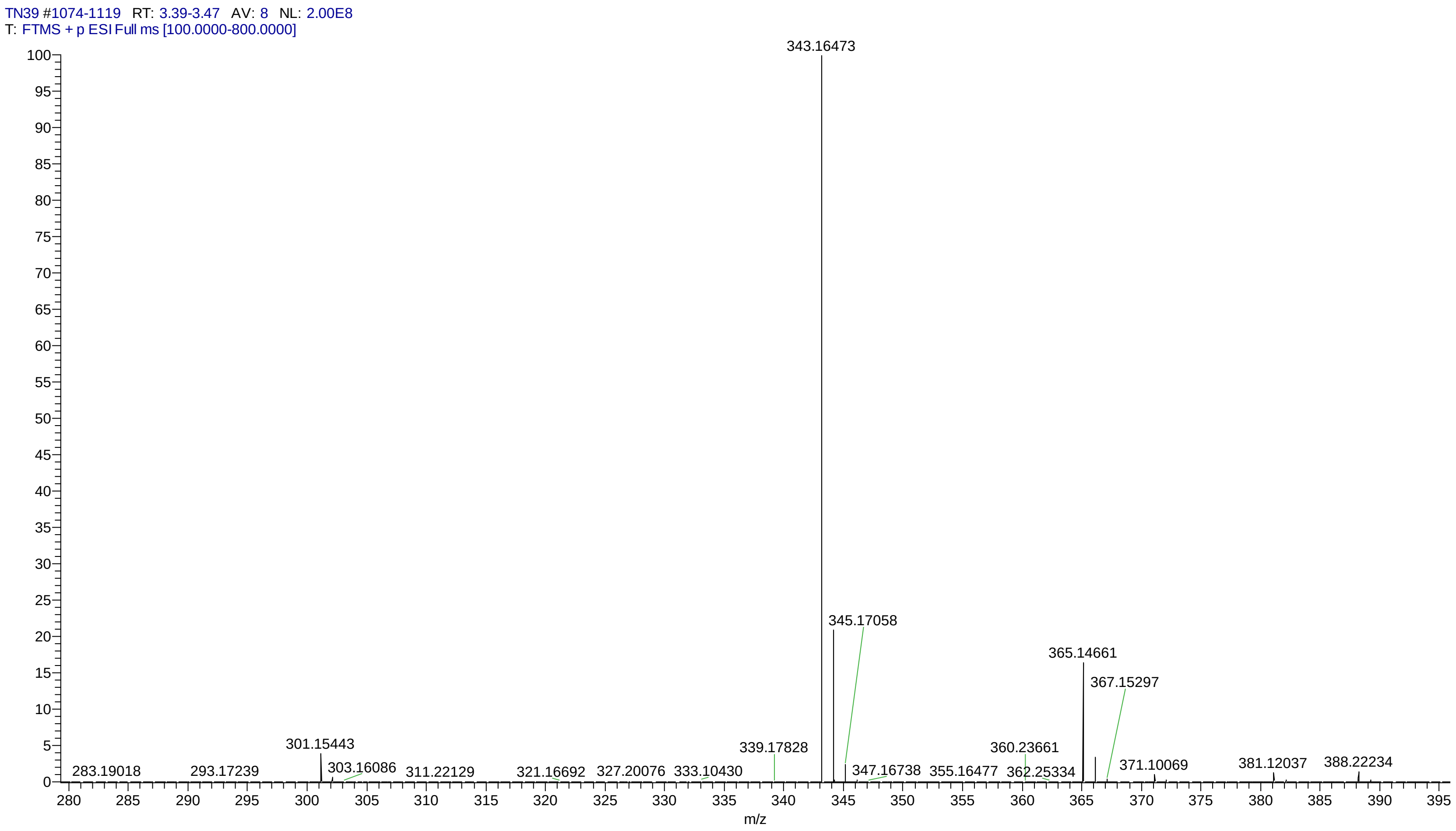

Figure S11: ^{1}H-NMR spectrum of compound **4b**

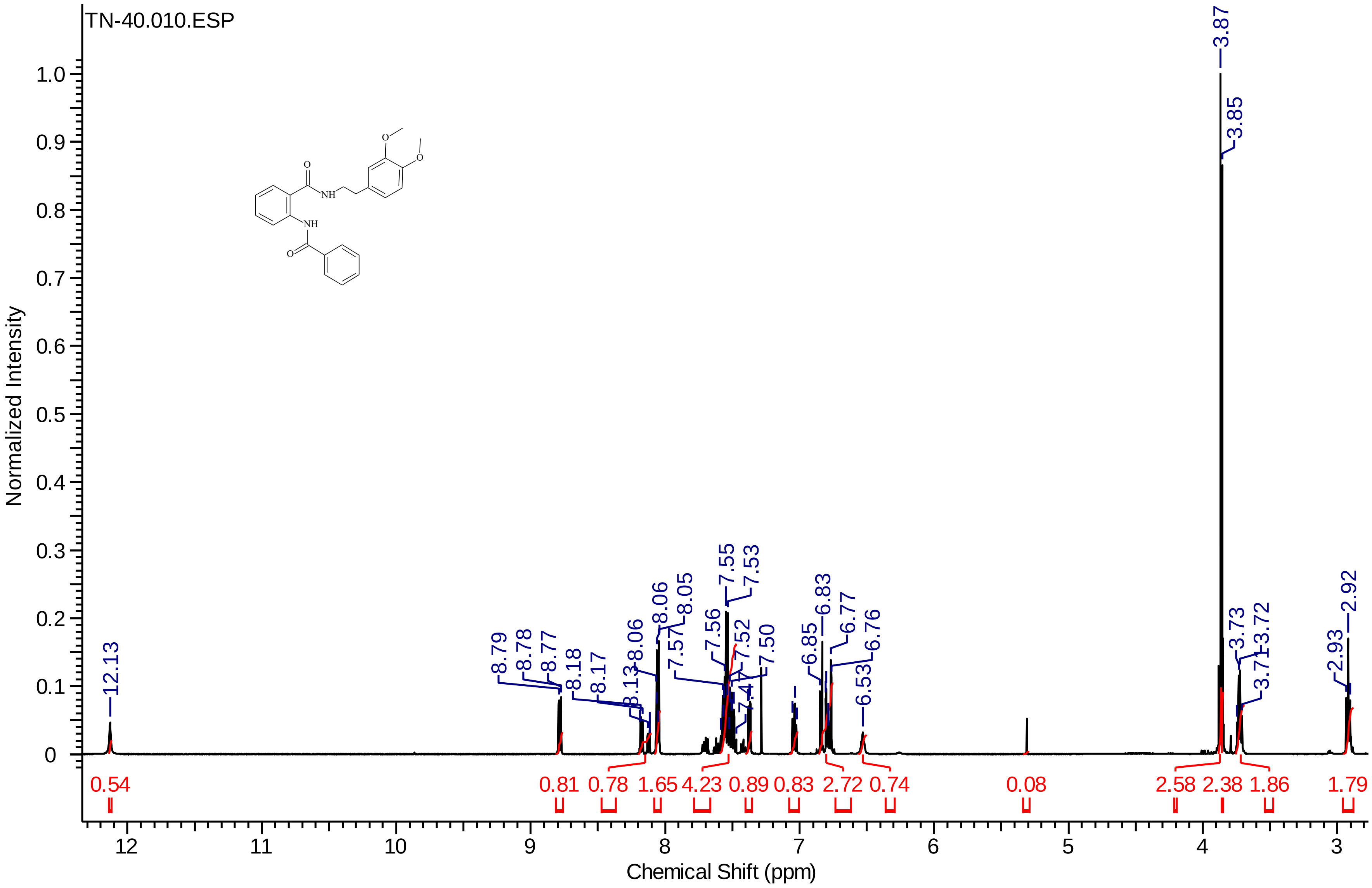

Figure S12: ^{13}C-NMR spectrum of compound **4b**

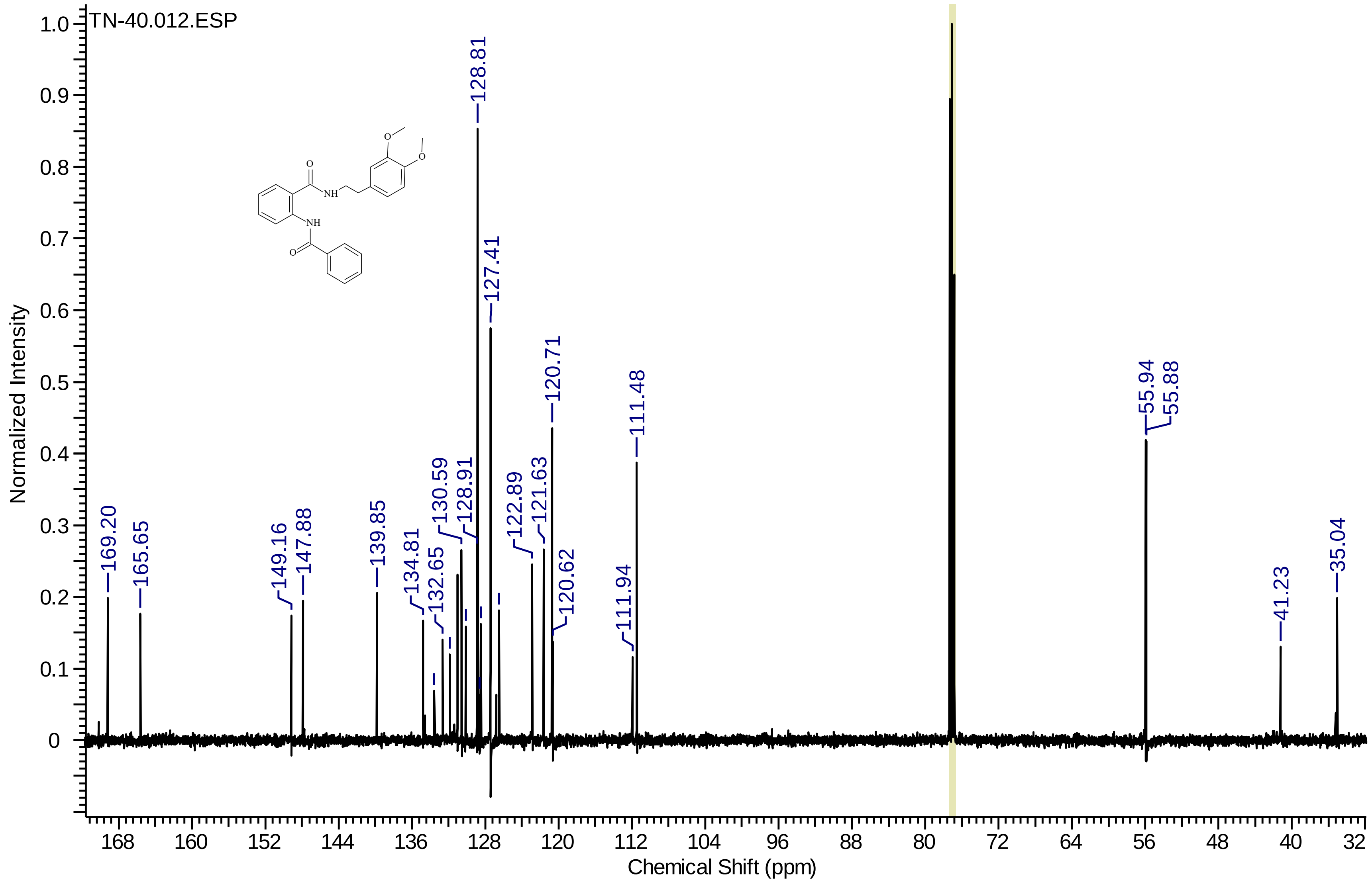

Figure S13: DEPT spectrum of compound **4b**

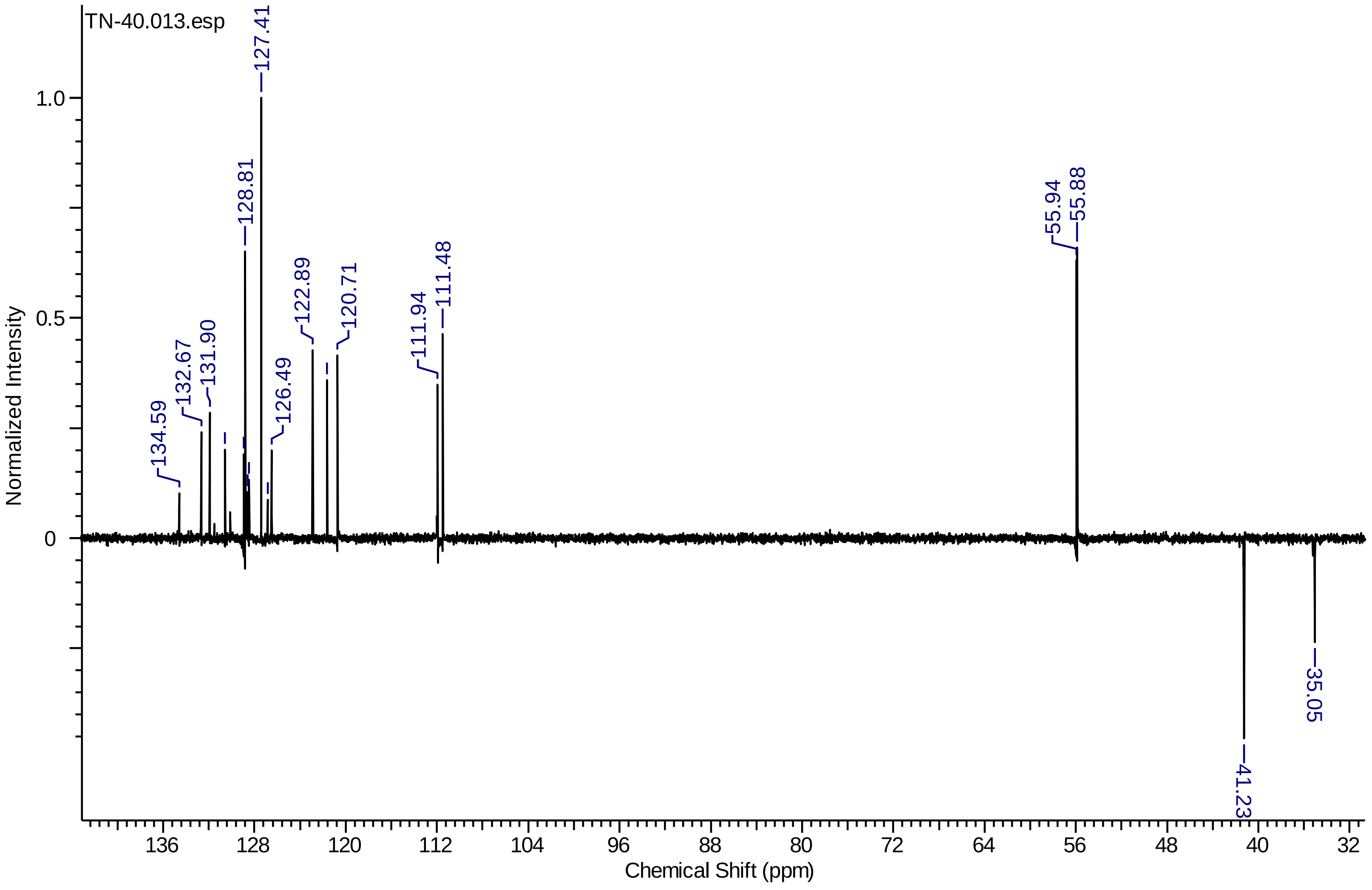

Figure S14: FT-IR spectrum of compound **4b**

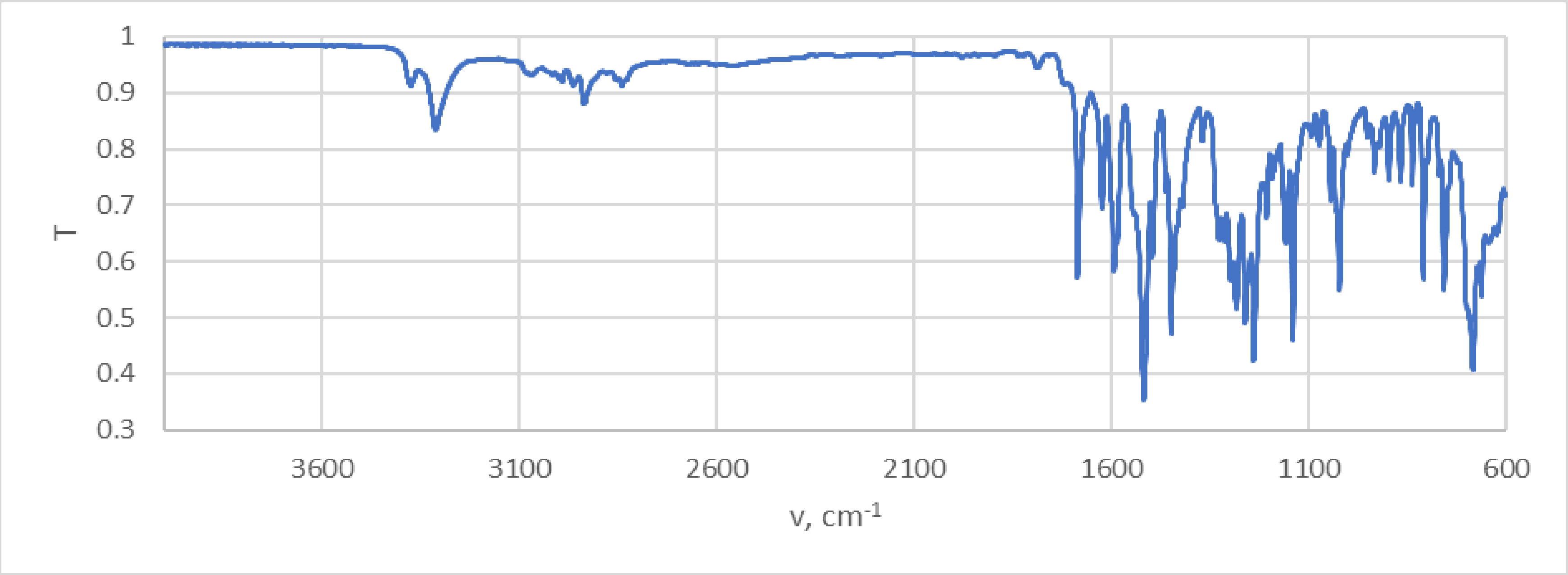

Figure S15: Mass spectrum of **4b.**

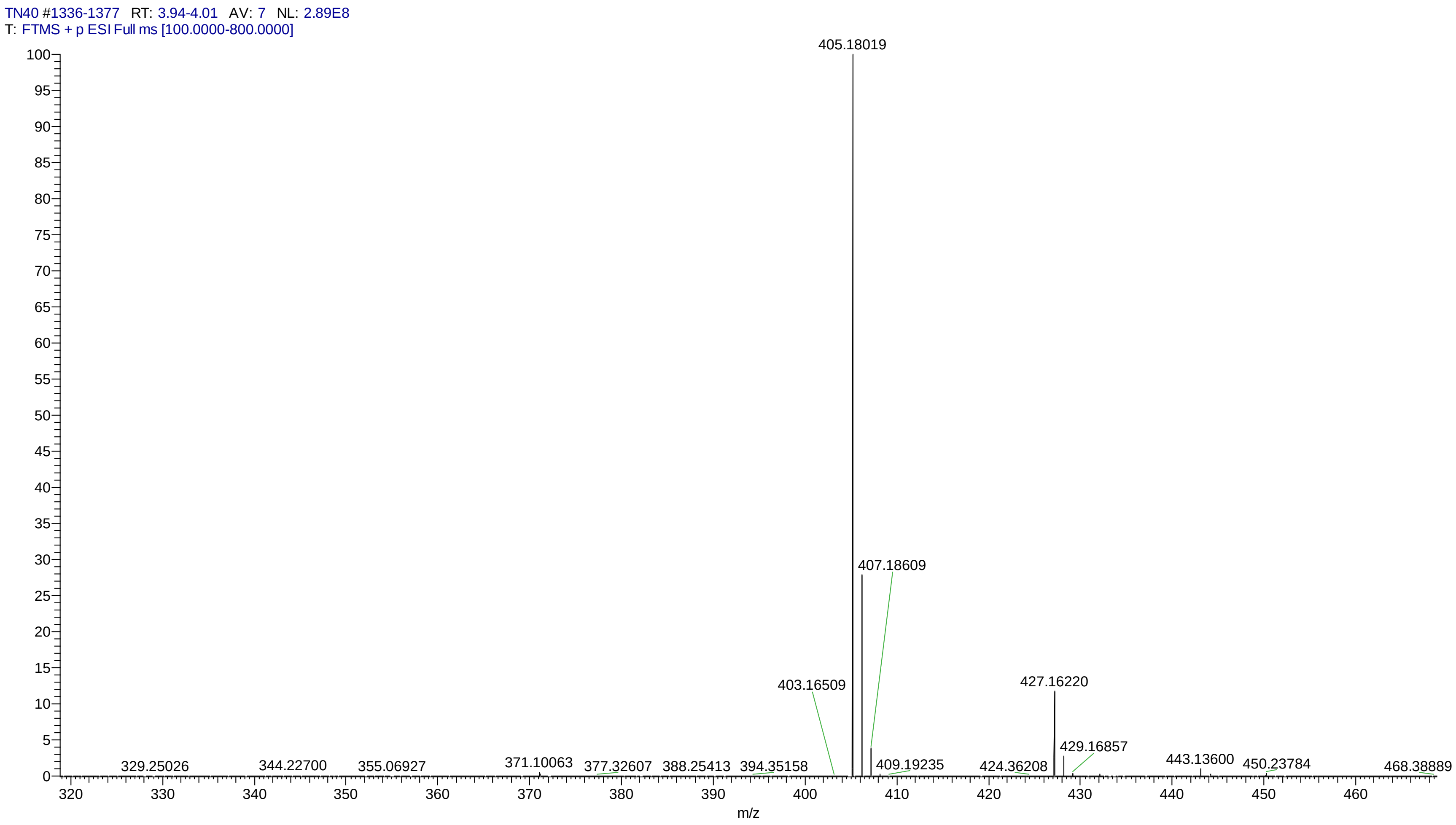

Figure S16: ^{1}H-NMR spectrum of compound **4c**

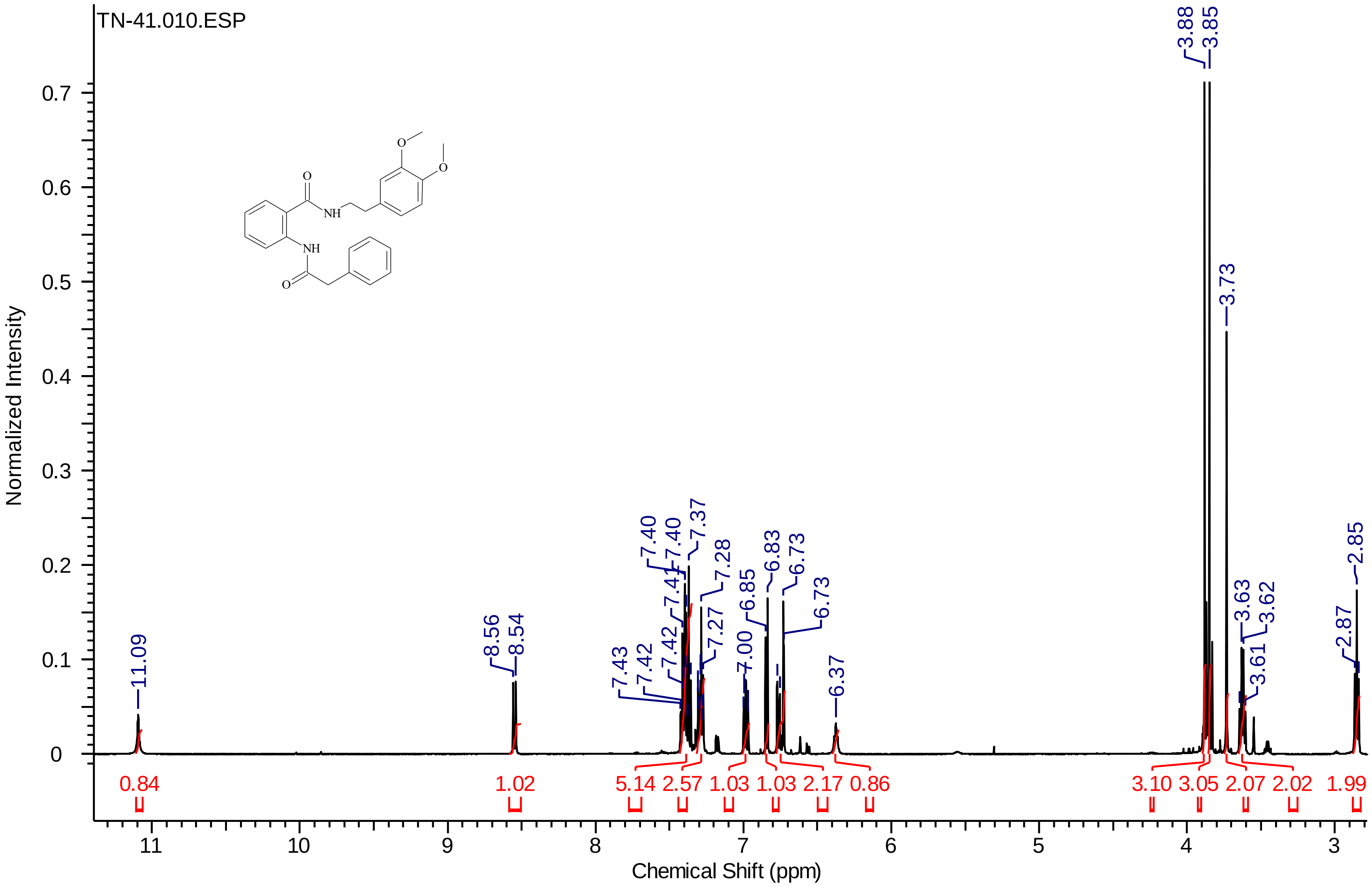

Figure S17: ^{13}C-NMR spectrum of compound **4c**

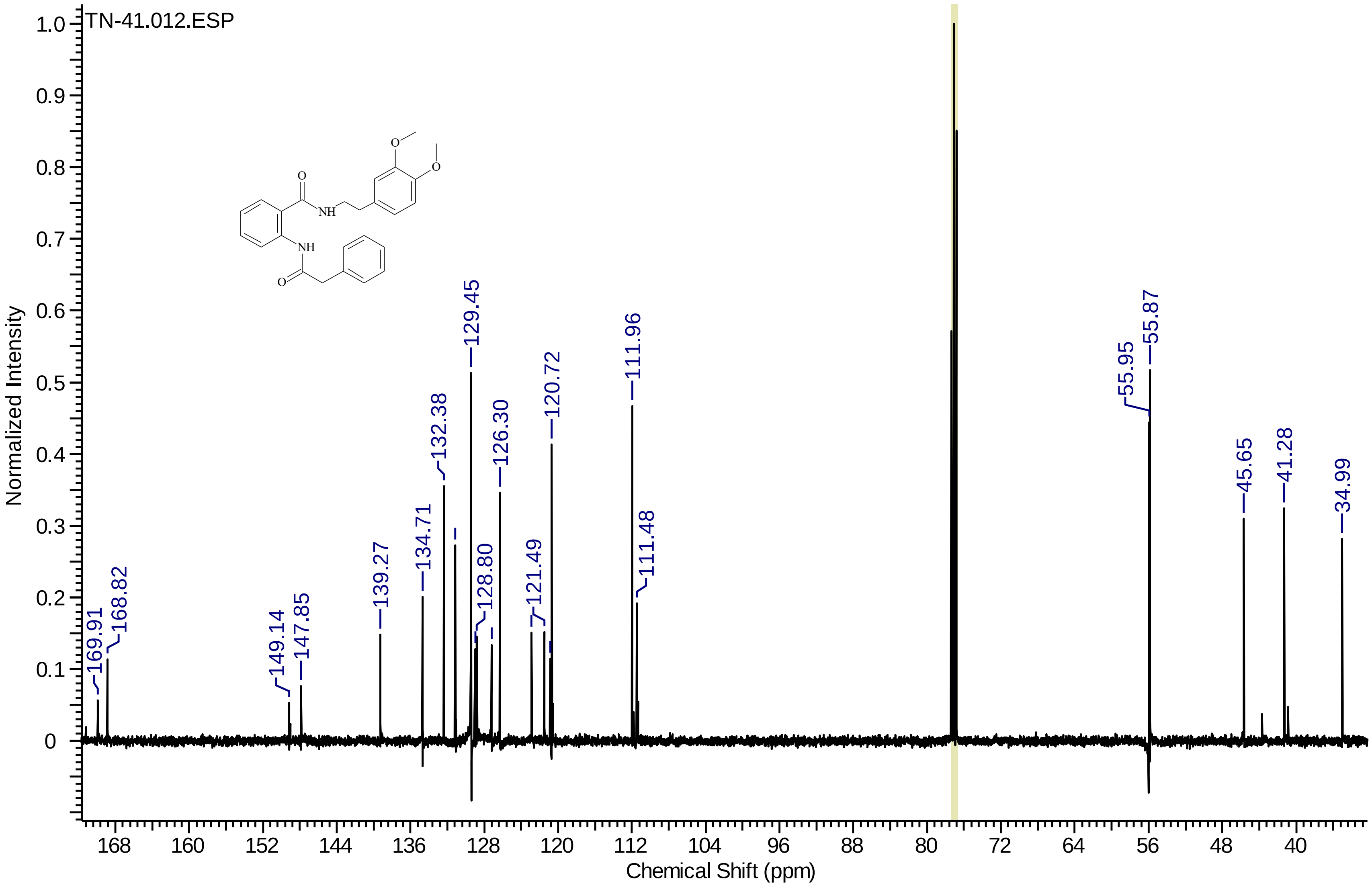

Figure S18: DEPT spectrum of compound **4c**

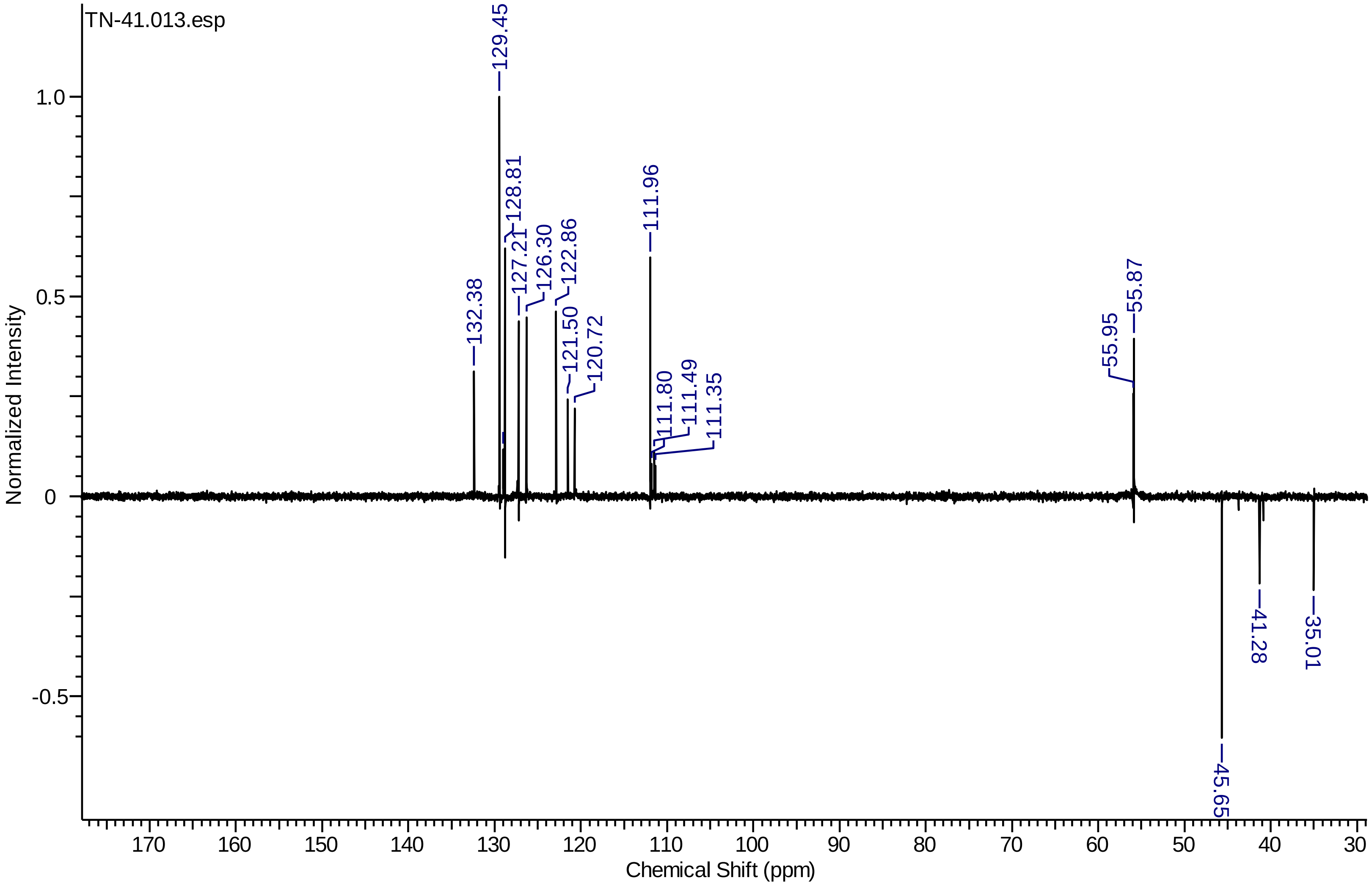

Figure S19: FT-IR spectrum of compound **4c**

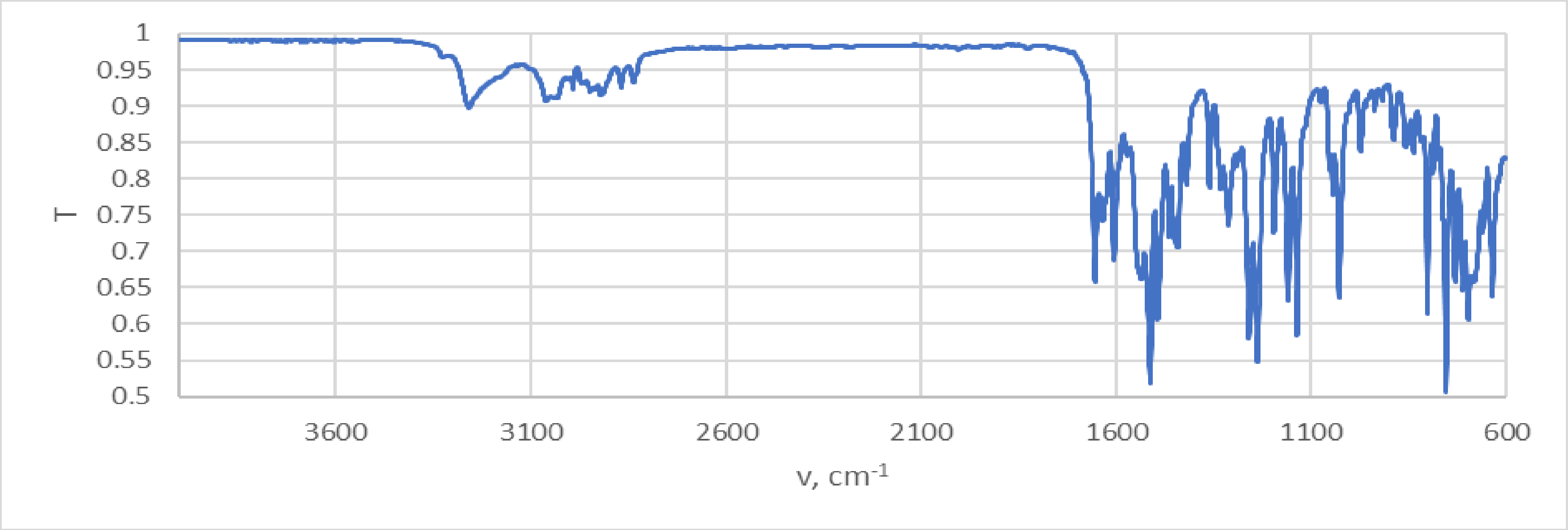

Figure S20: Mass spectrum of **4c.**

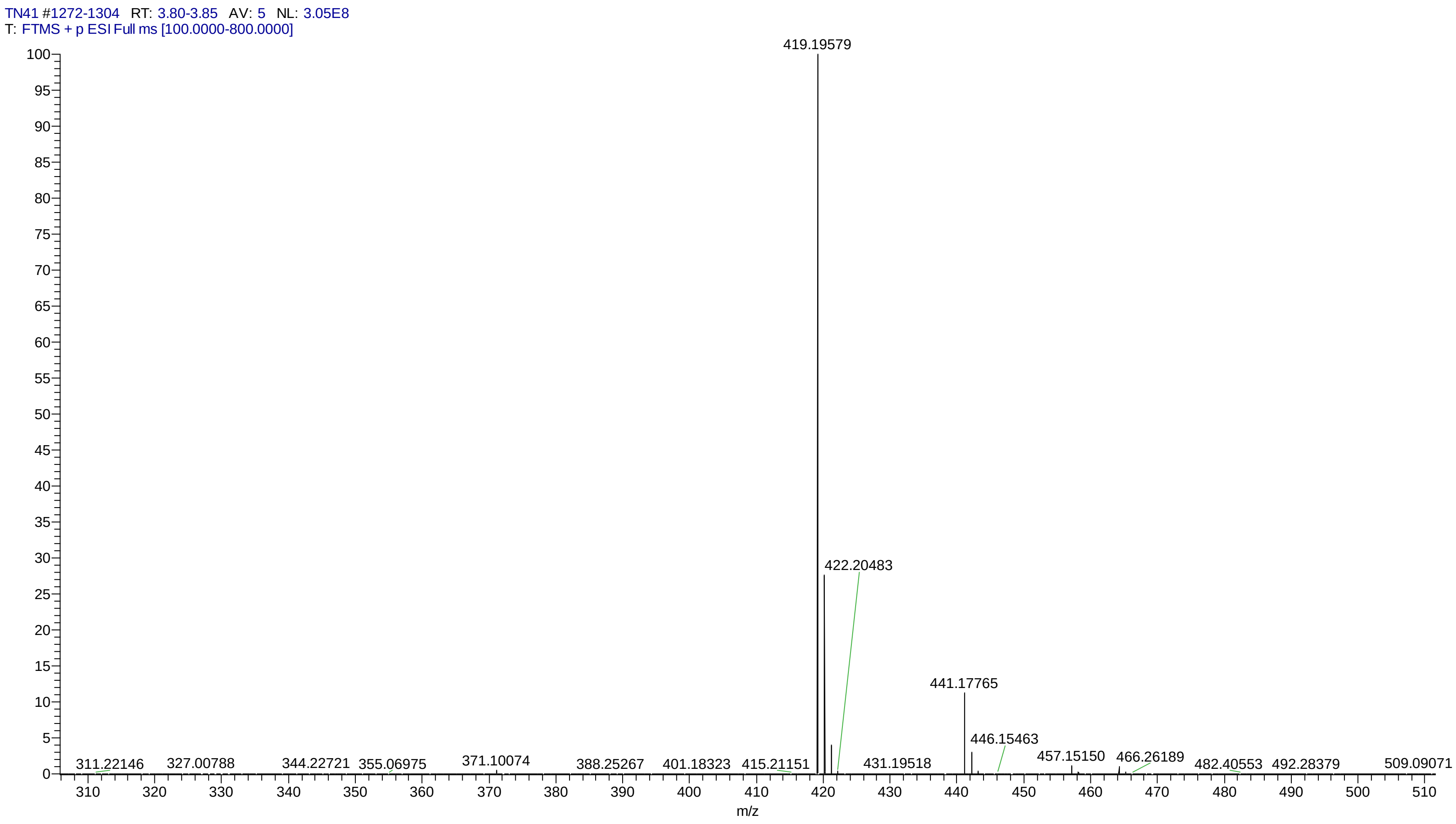

Figure S21: ¹H-NMR spectrum of compound **4d**

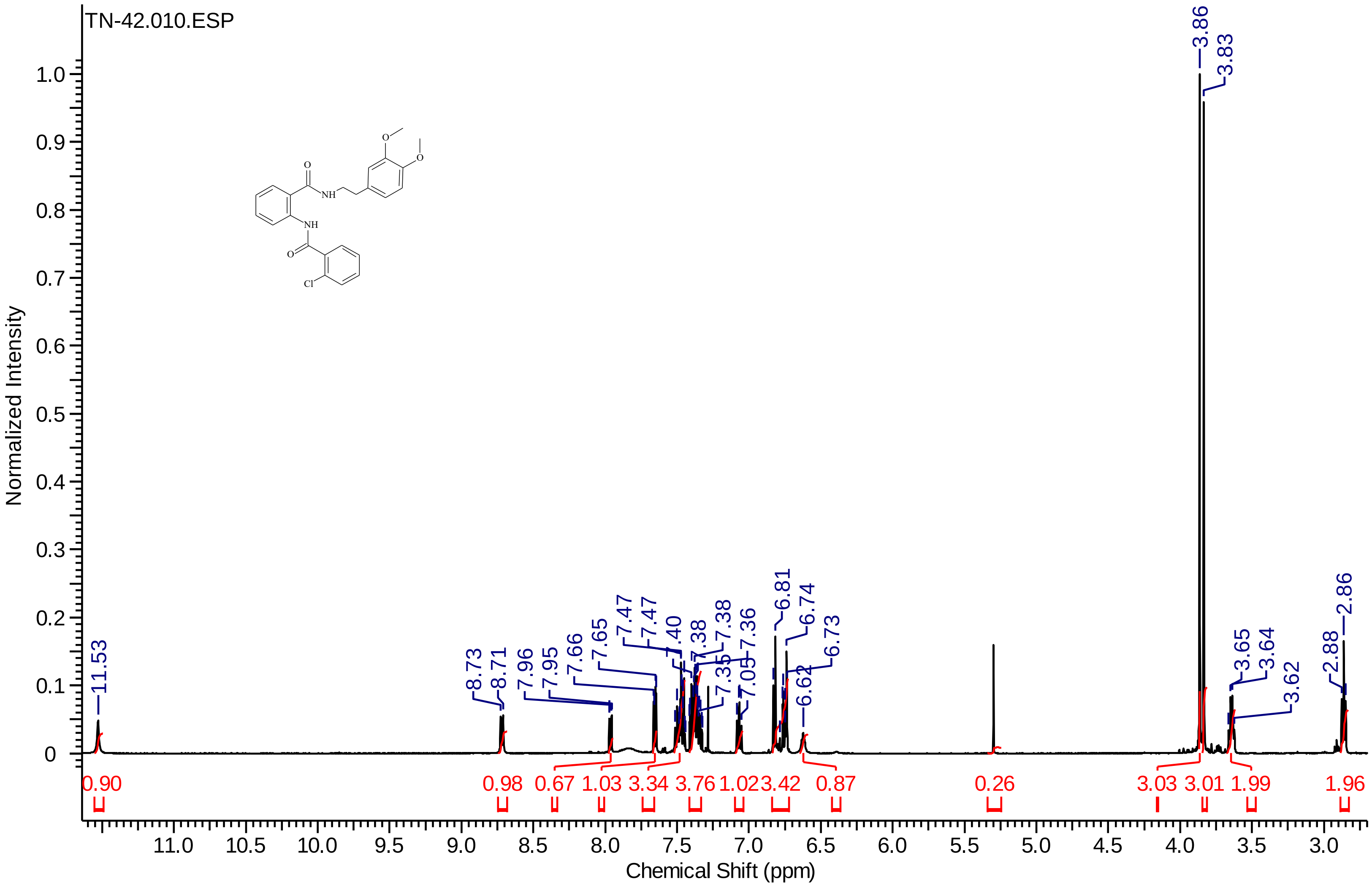

Figure S22: ^{13}C-NMR spectrum of compound **4d.**

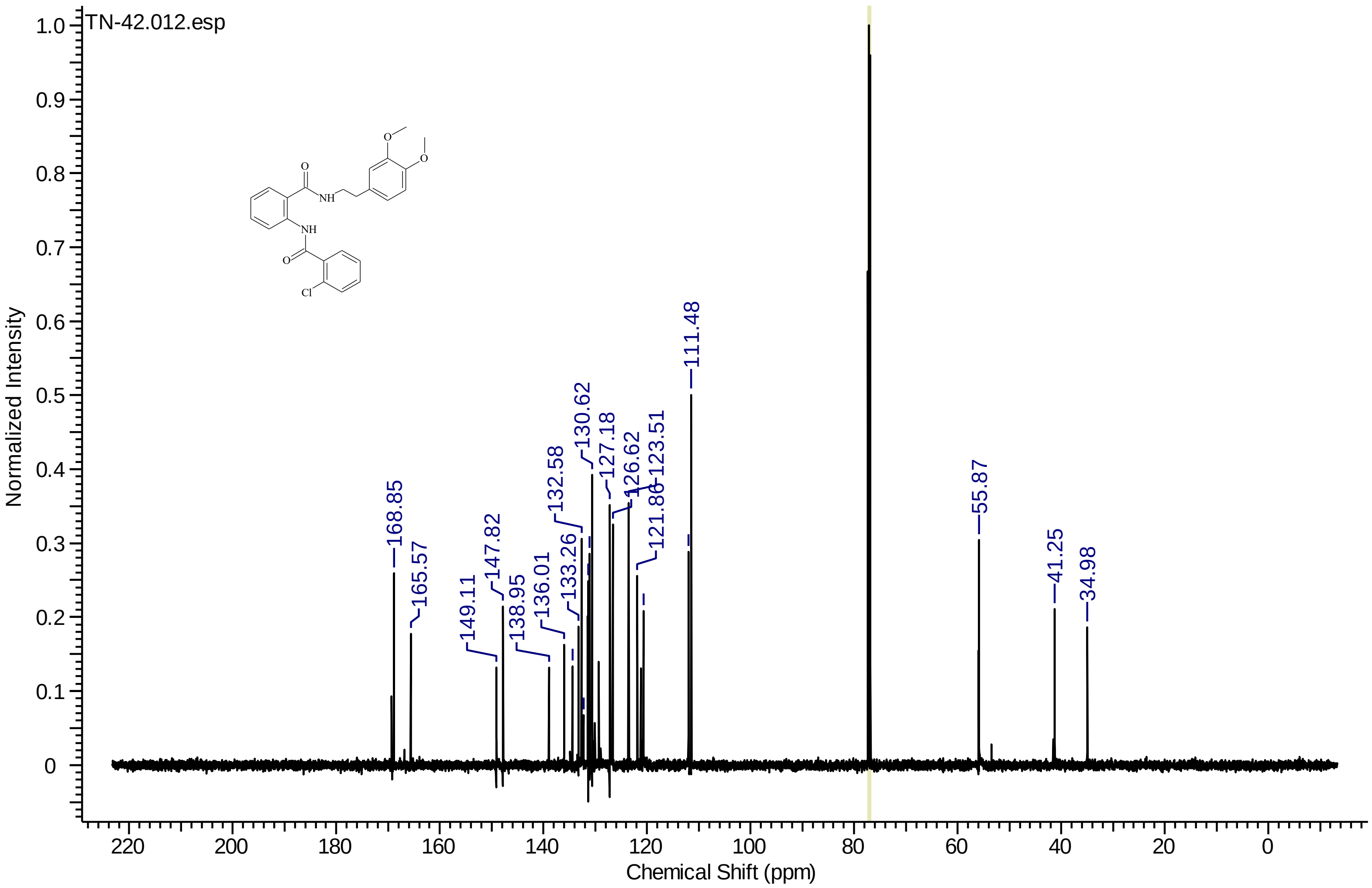

Figure S23: DEPT spectrum of compound **4d**

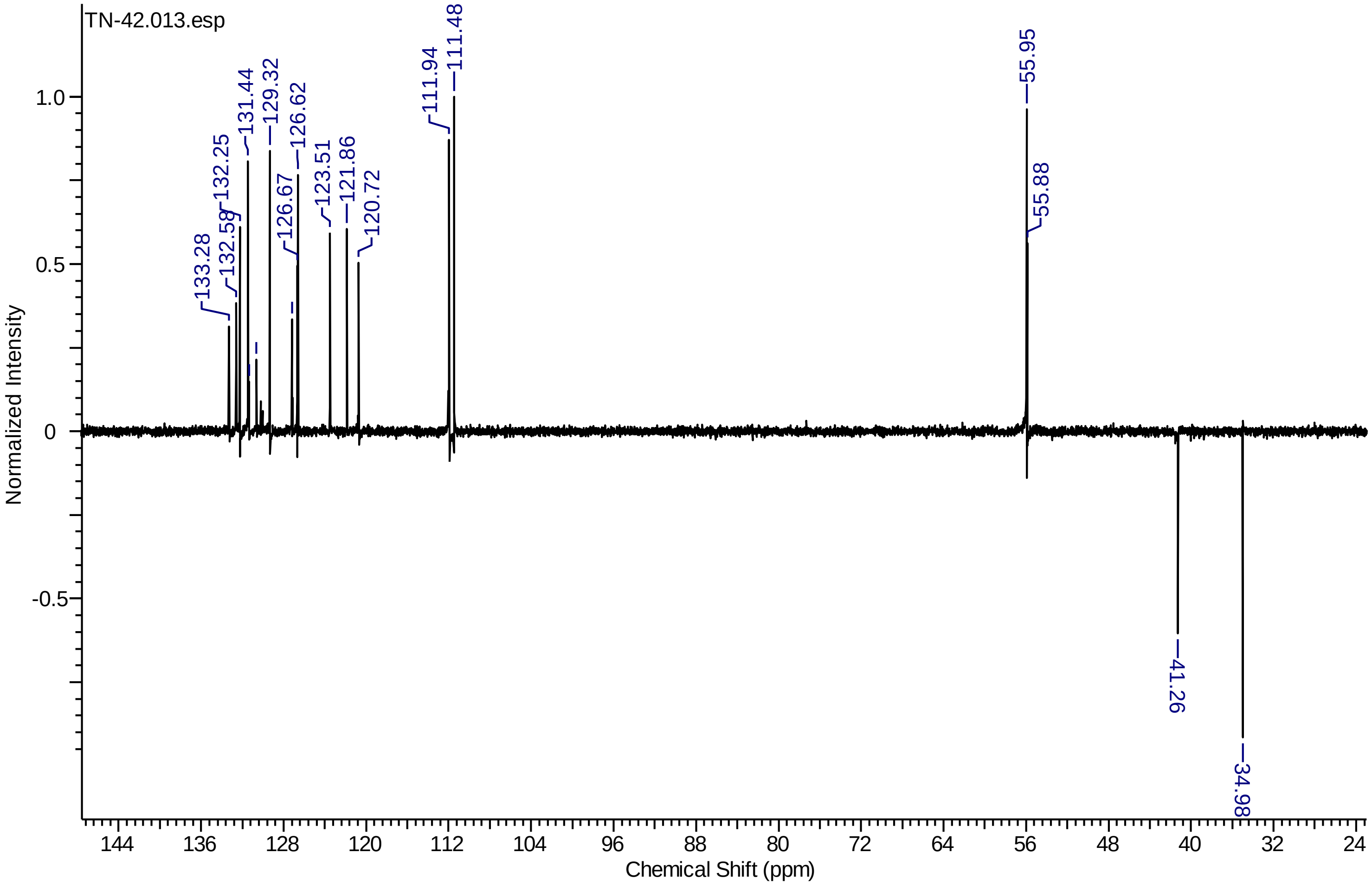

Figure S24: FT-IR spectrum of compound **4d**

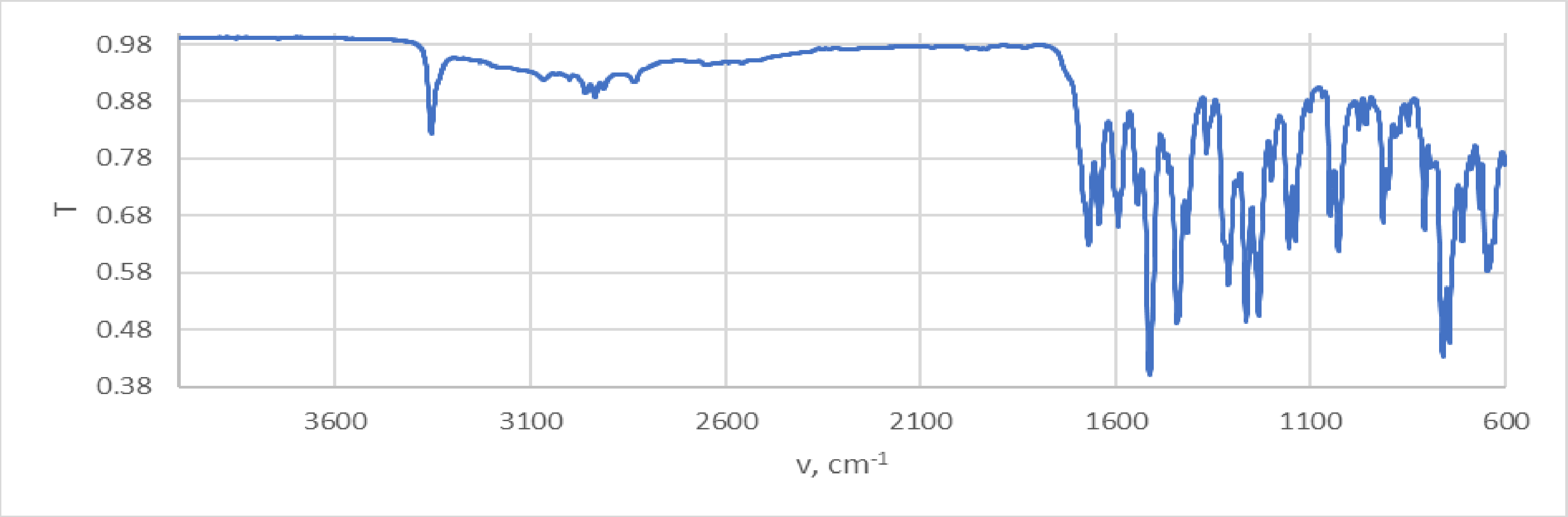

Figure S25: Mass spectrum of **4d.**

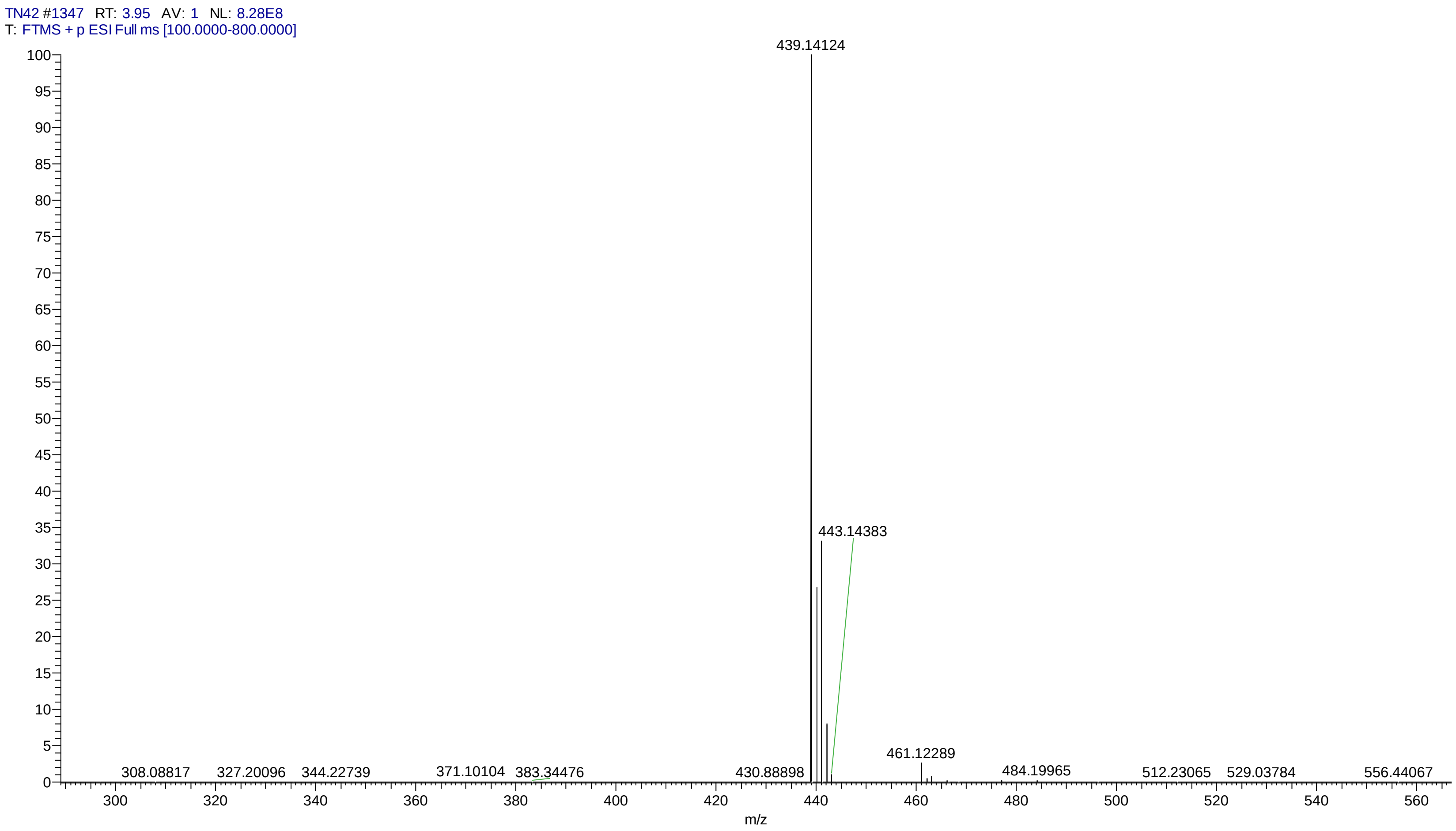

Figure S26: ^{1}H-NMR spectrum of compound **4e**

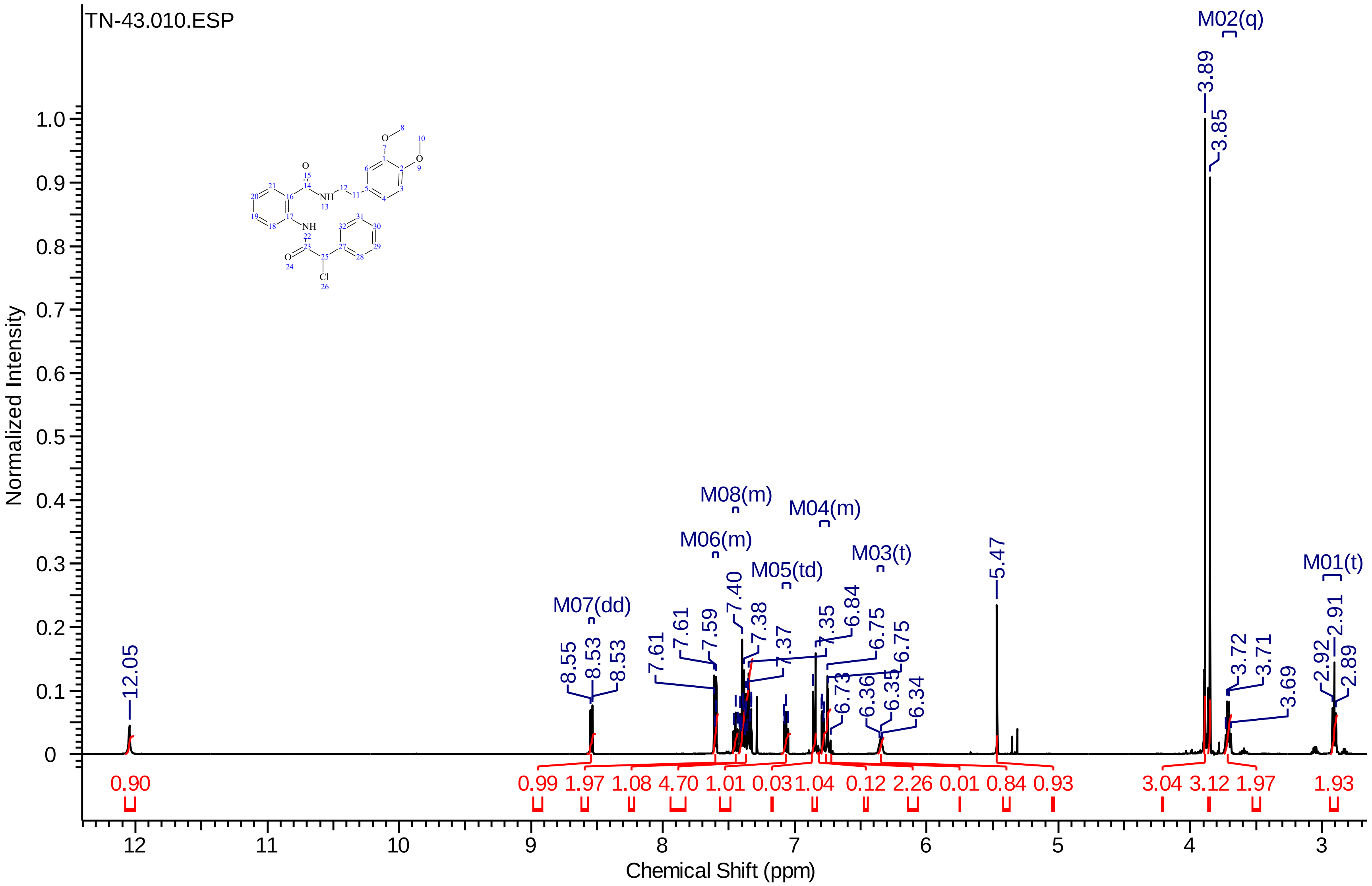

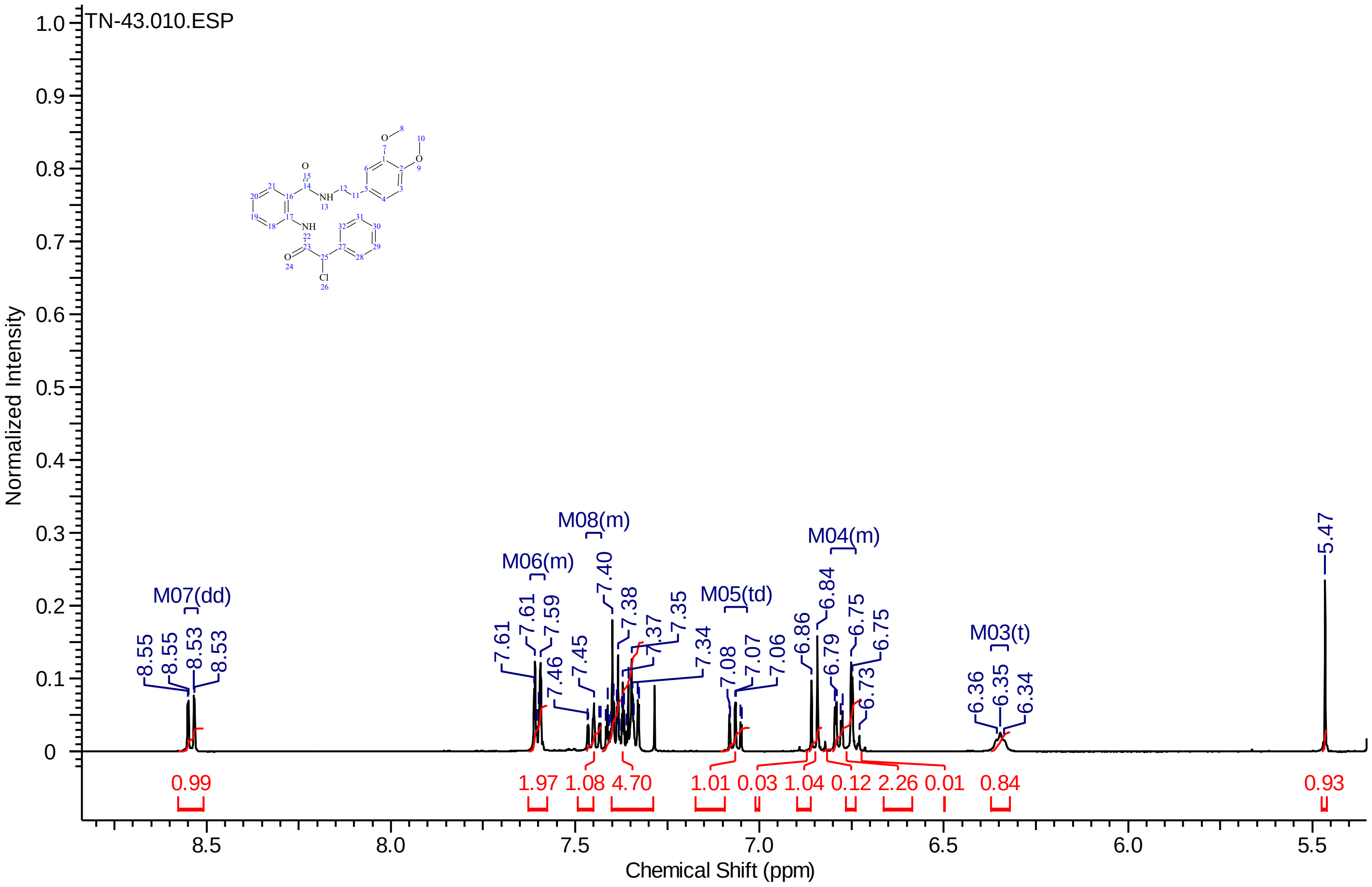

TN-43.010.ESP
Normalized Intensity
Chemical Shift (ppm)
M07(dd)
M06(m)
M08(m)
M05(td)
M04(m)
M03(t)
8.55
8.55
8.53
8.53
7.61
7.61
7.59
7.46
7.45
7.40
7.38
7.37
7.35
7.34
7.08
7.07
7.06
6.86
6.84
6.79
6.75
6.75
6.73
6.36
6.35
6.34
5.47
0.99
1.97
1.08
4.70
1.01
0.03
1.04
0.12
2.26
0.01
0.84
0.93

Figure S27: ¹³C-NMR spectrum of compound **4e**

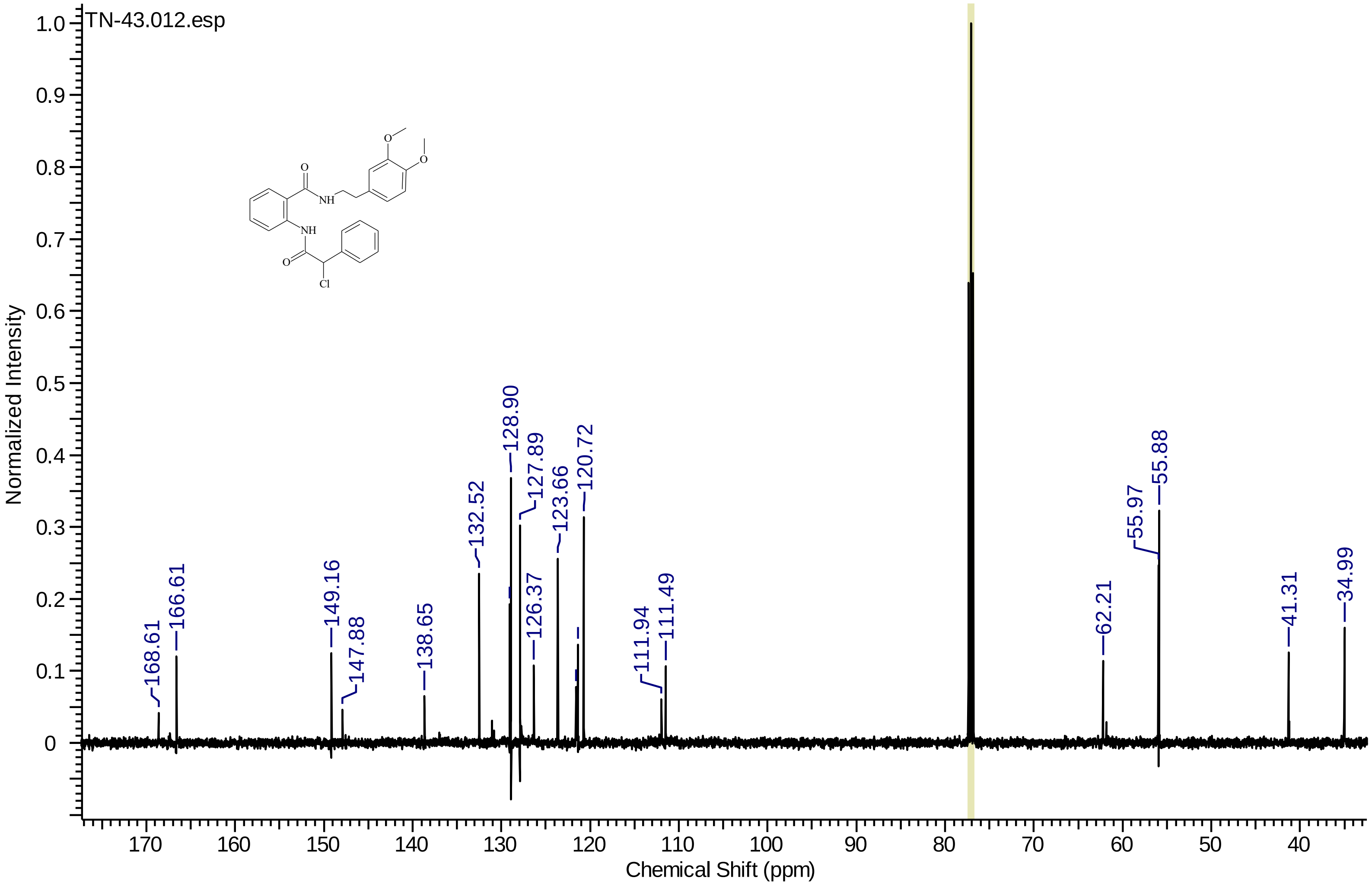

Figure S28: DEPT spectrum of compound **4e**

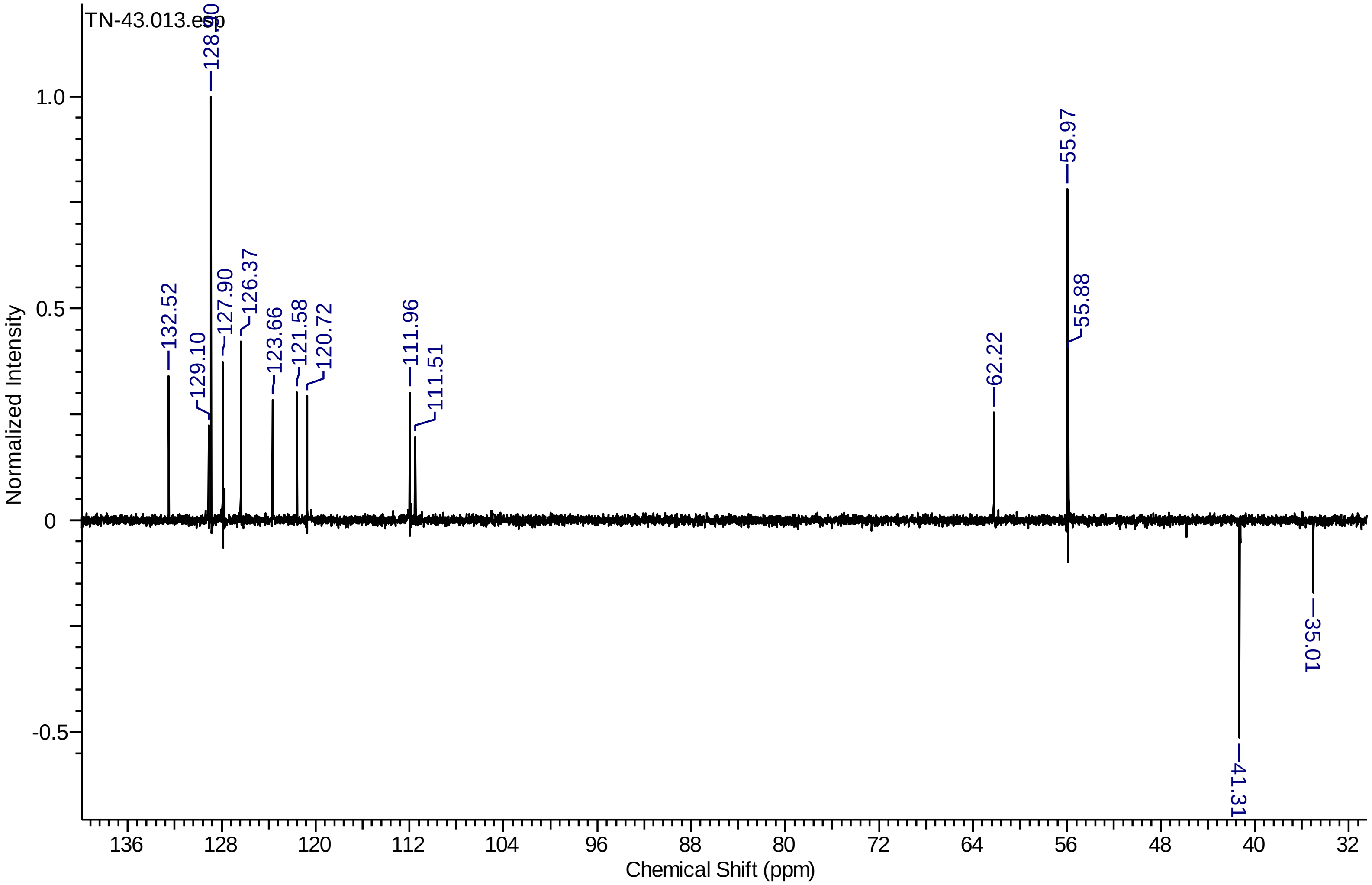

Figure S29: FT-IR spectrum of compound **4e**

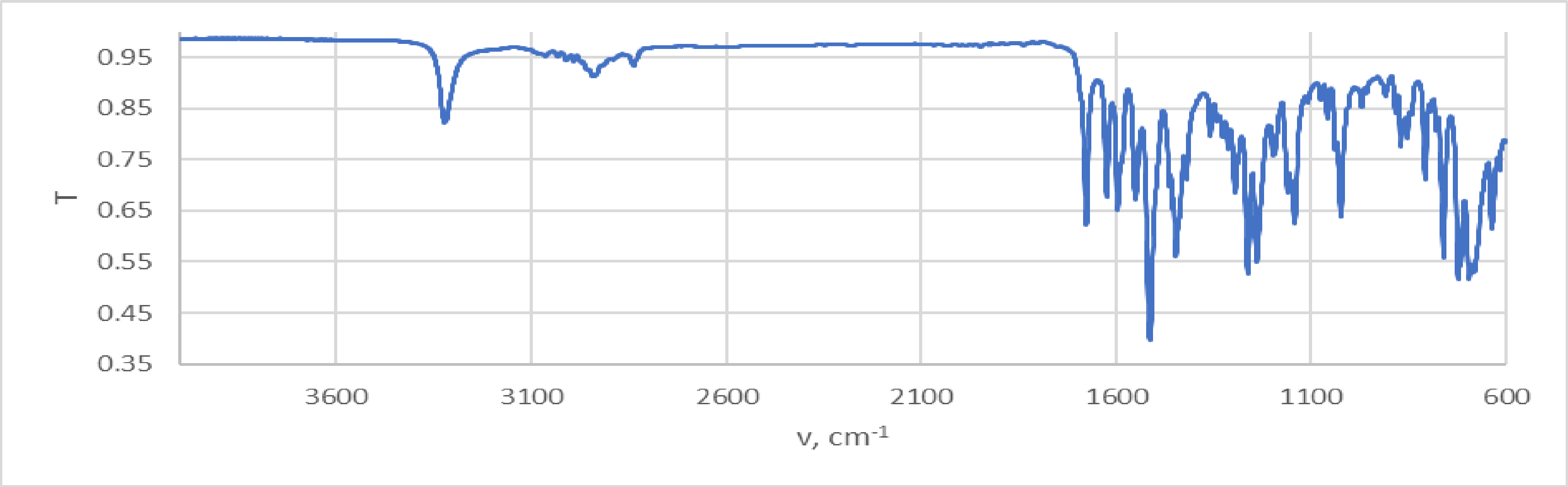

Figure S30: Mass spectrum of **4e.**

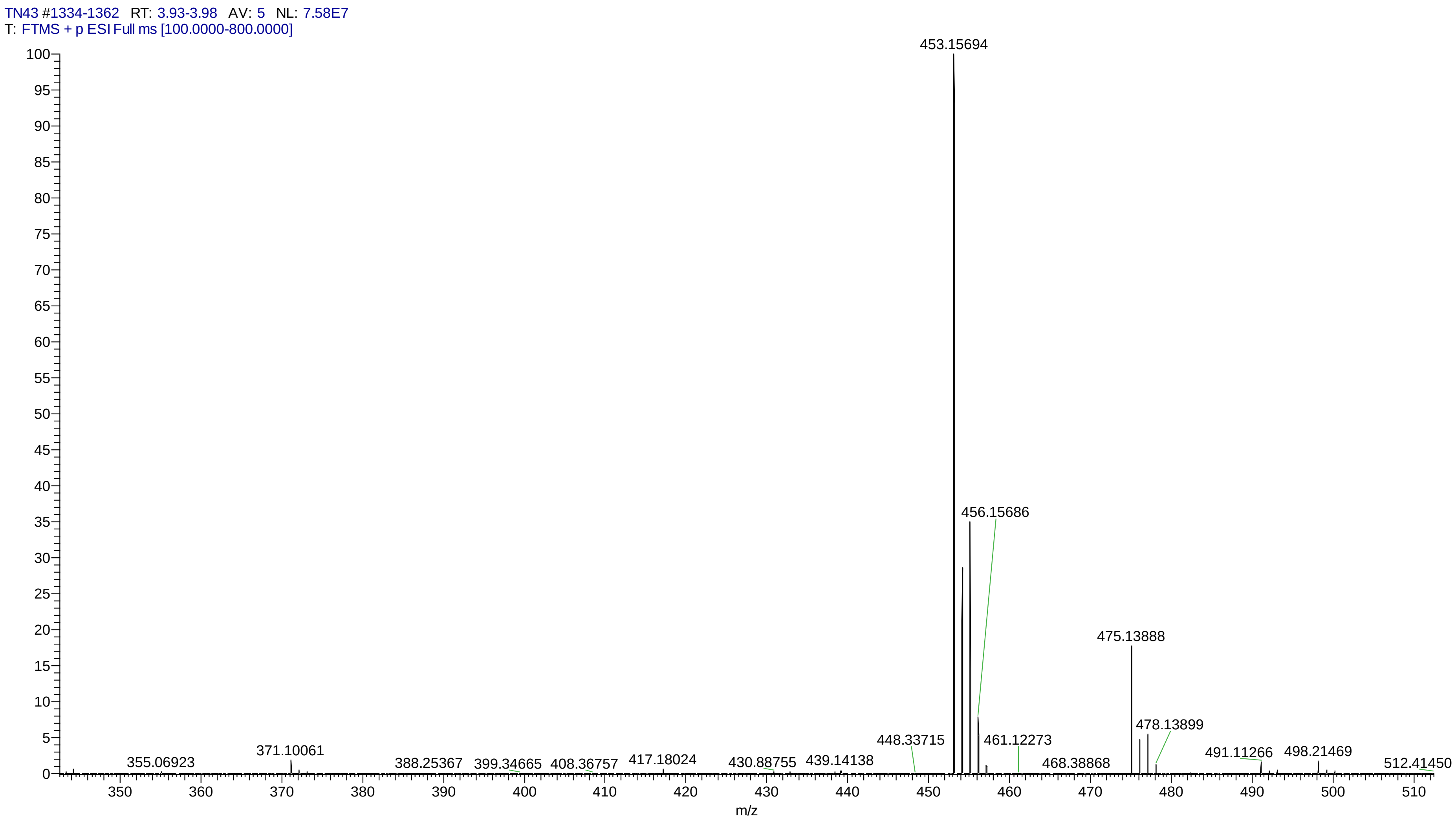

Supplement: Supplementary file 1 [file pharmaceuticals-16-01660-s001.zip › pharmaceuticals-2690355-supplementary.pdf]
